# Development of New Targeted Nanotherapy Combined with Magneto-Fluorescent Nanoparticles against Colorectal Cancer

**DOI:** 10.3390/ijms24076612

**Published:** 2023-04-01

**Authors:** Gonçalo A. Marcelo, David Montpeyó, Joana Galhano, Ramón Martínez-Máñez, José Luis Capelo-Martínez, Julia Lorenzo, Carlos Lodeiro, Elisabete Oliveira

**Affiliations:** 1 BIOSCOPE Group, LAQV@REQUIMTE, Chemistry Department, NOVA School of Science and Technology, 2829-516 Caparica, Portugal; 2 Institut de Biotecnologia i Biomedicina, Departament de Bioquímica i de Biologia Molecular, Universitat Autònoma de Barcelona, Bellaterra, 08193 Barcelona, Spain; 3 Instituto Interuniversitario de Investigación de Reconocimiento Molecular y Desarrollo Tecnológico, Universitat Politècnica de València, Universitat de València, 46022 Valencia, Spain; 4 PROTEOMASS Scientific Society, Rua dos Inventores, Madam Parque, Caparica Campus, 2825-182 Caparica, Portugal

**Keywords:** target nanocarriers, oral formulations, colorectal cancer, target drug delivery, targeted anticancer therapy

## Abstract

The need for non-invasive therapies capable of conserving drug efficiency and stability while having specific targetability against colorectal cancer (CRC), has made nanoparticles preferable vehicles and principal building blocks for the development of complex and multi-action anti-tumoral approaches. For that purpose, we herein report the production of a combinatory anti-tumoral nanotherapy using the production of a new targeting towards CRC lines. To do so, Magneto-fluorescent NANO3 nanoparticles were used as nanocarriers for a combination of the drugs doxorubicin (DOX) and ofloxacin (OFLO). NANO3 nanoparticles’ surface was modified with two different targeting agents, a newly synthesized (anti-CA IX acetazolamide derivative (AZM-SH)) and a commercially available (anti-epidermal growth factor receptor (EGFR), Cetuximab). The cytotoxicity revealed that only DOX-containing nanosystems showed significant and even competitive cytotoxicity when compared to that of free DOX. Interestingly, surface modification with AZM-SH promoted an increased cellular uptake in the HCT116 cell line, surpassing even those functionalized with Cetuximab. The results show that the new target has high potential to be used as a nanotherapy agent for CRC cells, surpassing commercial targets. As a proof-of-concept, an oral administration form of NANO3 systems was successfully combined with Eudragit^®^ enteric coating and studied under extreme conditions.

## 1. Introduction

Colorectal cancer (CRC) is the second deadliest cancer and the third most common malignancy worldwide [1]. Due to its characteristics, it can become highly aggressive and develop resistance to several therapeutic strategies [2,3]. All these aspects contribute to the alarming rising prevalence of CRC worldwide [4], making the development of more efficient anticancer therapies of utmost importance.

Nanoparticle-based materials have been widely developed and successfully employed as anticancer tools or as platforms to improve the efficacy of already known therapies, with metallic/metal-oxide nanoparticles oftentimes being used to this end [5,6]. Other nanosystems have also gained particular interest, specifically those containing mesoporous silica nanoparticles/structures, whose highly porous matrix renders them with unique cargo delivery capacities, surface manoeuvrability, targetability or even spectroscopical activity [7,8]. Amongst these, precision diagnosis and therapeutic systems are of particular interest, due to their increased performance and variability [9,10,11].

Specific characteristics of tumour-like environments, such as hypoxia and acidic extracellular pH, can thus be utilized as targets for specific delivery of nanoparticle-based systems. Cancer cell environments oftentimes present uncontrolled growth that results in improperly structured and functioning vasculature, low oxygenation, and upregulation of homeostasis-associated enzymes, such as carbonic anhydrases (CAs, EC 4.2.1.1) [12].

CAs are ubiquitous metalloenzymes that catalyse the simple physiological hydration of carbon dioxide to bicarbonate ions and protons (CO_2_ + H_2_O <-> HCO^3−^ + H^+^), with most containing in their active centre a zinc ion (Zn^2+^), which is essential for catalysis. Besides regulation of pH and CO_2_ homeostasis, CAs are also responsible for CO_2_ and HCO^3−^ transport between metabolizing tissues and lungs, electrolytes secretion, some biosynthetic processes (i.e., glucogenesis, lipogenesis and urea genesis), bone resorption, and tumorigenicity [12]. Their broad activity spectrum arises from the 15 presently known isozymes, with CAs I, II, III, VII and XIII residing in the cytosol; CAs IV, IX, XII, XIV, and XV being associated with membranes; CAs VA and VB occurring in mitochondria; and CA VI existing only as a secreted isoform [13].

The occurrence of an elevated number of CA isoforms in cellular membranes of numerous organs and the association of their overexpressed activity to some types of tumours have thus sparked a special interest in CAs as potential targets for tumour diagnosis and therapy [14,15]. The human (h)CA IX is an example of a tumour-associated CA isoform, with several groups associating it with tumour activity, cell proliferation, cell adhesion and malignant cell invasion [16,17], being thus also commonly considered as a potential biomarker for tumour diagnosis [18]. More specifically, hCA IX’s overexpression levels and increased activity have been recently associated with higher rates of hypoxia survival and cell invasion in tumours [19,20].

The inhibition of CAs has been increasingly reported as a potential pathway for cancer therapy by using CA-directed pharmacological agents as CAs inhibitors, capable of imparting CAs activity and normal cell mechanisms [21,22]. Among others, 5-acetamido-1,3,4,-thiadiazole-2-sulfonamide, commonly known as Acetazolamide (AZM) and its analogues, have exhibited outstanding and selective inhibitory activities towards CAs, being extensively used clinically as diuretics, anti-glaucoma, or anti-convulsant agents [23,24,25]. Regarding anti-tumoral therapies, specific CA and hCA IX inhibitors have also been reported as potential hypoxia-activable inhibitors and targeting agents [14,26,27,28].

The development of novel inhibitors capable of targeting CAs, specifically hCA IX, is thus of high interest, especially if they can be conjugated with pro-drugs or with nanometric vectors, rendering them targetable to the overexpressed hCA IX in cancerous environments.

Associated with CA IX action and overexpression in hypoxic and invasive tumours, such as CRC [18,19], is also the commonly overexpressed epithelial growth factor receptor (EGFR), which mediates cancer initiation, progression and proliferation [29,30]. This association makes anti-EGFR agents also good targeting probes, with several works reporting the conjugated use of several anti-EGFR antibodies, such as the commercial Cetuximab (CTX), with therapeutic molecules (drugs, pro-drugs, etc.) and nanometric vectors for either diagnosis or therapeutics in CRC [31,32,33].

Considering this, herein we report the synthesis of a novel AZM-derivative containing a sulfhydryl anchoring moiety, and its subsequent biofunctionalization on superparamagnetic, fluorescent iron oxide-cored mesoporous silica nanoparticles (NANO3-SH). This system was successfully evaluated as a potential anticancer tool against CRC cell lines [34]. The development of a dual anti-EGFR/anti-hCA IX (CTX+AZM) targeting nanoprobe is also reported, and its performance as a precision therapeutic tool against CRC is compared with that of each individual NANO3-anchored antigen.

Nonetheless, the method of administration of drugs for therapeutic purposes is one critical step in the development of new ground-breaking therapies. The unsuccessful accounting of possible complex interactions with the body’s solid and liquid interfaces and all related arising hindrances dictate the successful rate of said therapies, even for those showing outstanding performances in silico/in vitro. Not only that, but with the ever-growing pursuit of non-invasive techniques, orally administered and inhalation-based products have gained particular interest in pharmaceutical industries/markets [35,36].

These points are particularly familiar to CRC, which, due to its body location, sees many oral delivery therapies being developed [37,38,39], of which some might be withdrawn from clinical usage. Most withdrawals are not only to problems of stability when in contact with body fluids constituents, pH and temperature, but also to problems of long circulation instability and peripheral body accumulation in organs/members other that the colon [35,40]. Some of the major barriers to the development of an orally administered therapy against CRC are the passage time, intrinsic high absorption, and extreme pH conditions of the whole gastrointestinal tract [40]. Its success goes, thus, through the protection of the active compound of interest with an external resistant, but also manoeuvrable, agent. Such agents must be capable of both resisting extreme denaturing conditions and having a stimuli-responsive behaviour (i.e., pH, time, etc.) to correctly dispense the therapeutic compound at the targeted site/time [41]. For anti-CRC applications, the selected formulation must be capable of bypassing stomach extreme conditions and then small intestine absorption, before reaching the colon. For that purpose, in a proof-of-concept, NANO3-SH systems were protected with an enteric coating formulation of Eudragit^®^ polymers (PMMA-MMA copolymers), which showed a prominent capacity to effectively deliver the nanomaterials herein developed.

## 2. Results and Discussion

### 2.1. Synthesis of AZM-SH and Anti-CAIX Action

The potential of acetazolamide (AZM) and its derivatives to serve as selective vehicles for the delivery of cancer-specific drugs and their inhibition activity over cancer-related CAs has been hinted at in several works, with special attention being given to the hCA IX isoform [15,42,43]. With this in consideration, herein we propose the synthesis of a thiol moiety containing an AZM-derivative, as a possible hCA (CA IX included) inhibitor and targeting agent in the delivery of anticancer drugs. The interest in the inclusion of a terminal thiol to the original structure of AZM arises from the potential formation of disulphide bonds with anticancer drugs of interest, or with nanovectors with intrinsic anti-tumoral activity or with drug-delivery capacity, for targeted anticancer therapy.

**Scheme 1:**
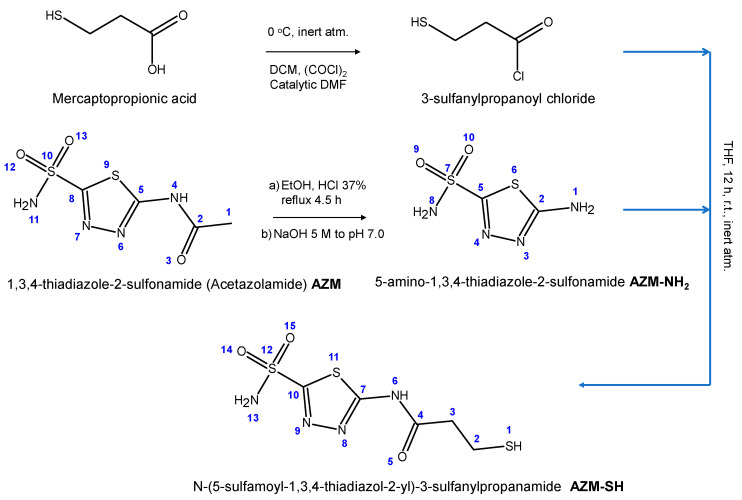
Synthetic workflow on the preparation of N-(5-sulfamoyl-1,3,4-thiadiazol-2-yl)-3-sulfanylpropanamide (AZM-SH) salt. Numbering system of atom (blue) used for the assignment of ^1^H and ^13^C NMR chemical shifts.

To synthesise the new compound, the amide in AZM was firstly hydrolysed to produce an available terminal NH_2_ group other than that of the sulfonamide moiety. By activation of the carboxylic acid moiety of the 3-mercaptopropionic acid, via Vilsmeier mechanism, and reaction with compound AZM-NH_2_, the new amide derivative (AZM-SH) was obtained. Intermediate AZM-NH_2_ and final product AZM-SH were characterised accordingly and compared to the initial AZM (Scheme 1).

**Figure 1:**
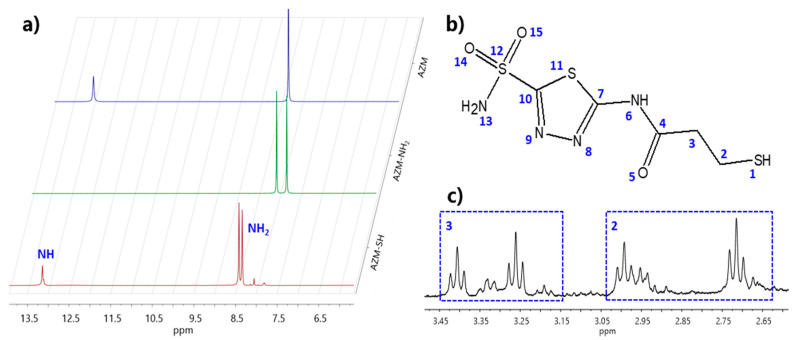
(**a**) Stacked ^1^H NMR spectra of AZM, AZM-NH_2_ AZM-SH (DMSO-d_6_, 400 MHz), for δ between 6 and 14 ppm. (**b**) AZM-SH structure and atom number assignment. (**c**) Close-up of the ^1^H NMR spectrum of AZM-SH (CD_3_CN, 400 MHz) and chemical shift assignment of sulfanylpropanamide C(2) and C(3) protons, seen as two crossed doublet-triplet (dt) signals.

The ^1^H and ^13^C NMR spectra of AZM-NH_2_ (Figure S1) confirm the total hydrolysis of the amide bond in AZM. This step is characterised by the disappearance of the 2.25 ppm -CH_3_ signal in the AZM ^1^H spectrum (Figure S2) and a right shift of the 13 ppm signal to 8.05 ppm, corresponding to the transformation of the secondary amide moiety into the terminal primary amine in AZM-NH_2_. Moreover, the loss of the acetamide partition of AZM was verified with the vanishing of its correspondent 22.83 ppm and 161.57 ppm signals in the ^13^C NMR spectrum of AZM-NH_2_. The successful binding of 3-sulfanylpropanoyl chloride to the amine (1) of AZM-NH_2_ was confirmed by ^1^H and ^13^C NMR spectra of AZM-SH, in DMSO-d6 and CD_3_CN (Figure S3). The reconstitution of the >NH signal at 13.13 ppm (DMSO-d_6_) and the surgency of a doublet at 8.38 ppm (DMSO-d_6_), corresponding to the sulfonamide -NH_2_, in the ^1^H NMR spectrum of the product (Figure S3), are the first hints at the successful synthesis of AZM-SH (Figure 1a). These signals are in accordance with the chemical shifts of the same groups in AZM’s NMR spectrum (Figure S1) and with those described for other thiadiazole-derived ligands [44,45].

**Figure 2:**
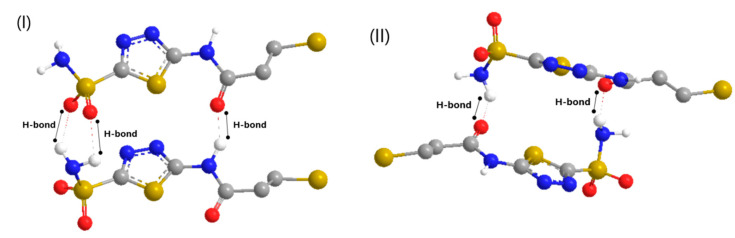
Suggested (**I**) head-head and (**II**) head-tail dimeric conformations of AZM-SH attained via hydrogen bonding.

Although not perceptible in the DMSO-d_6_ ^1^H NMR of AZM-SH, in CD_3_Cl, the signals relating the sulfanylpropanamide (NHCO-CH_2_-CH_2_-SH) C(2) and C(3) protons are visible between 3.45 and 3.15 ppm and between 3.05 and 2.65 ppm as a set of two doublet–triplet-like signals [46], thus supporting the successful synthesis of AZM-SH, by the inclusion of 3-sulfanylpropanoyl via amide bond formation. The obtained chemical shifts and crossings of the signals of C(2) and C(3) protons have been pointed out before, by Schuetze et al. [47], to arise from a certain degree of dimerization of the 3-sulfanylpropanoyl segment of the molecule via a disulphide bond (Figure 1c). The dimerization of the molecule is also suggested by the splitting of the -NH_2_ signal at 8.38 ppm, J = 24.4 Hz (Figure 1a), comprising the asymmetry created by intermolecular hydrogen bonding between the >NH/-NH_2_ and -C=O moieties of adjacent molecules (Figure 2), similarly to those formed in some reported AZM dimers [48,49].

Complete conversion of AZM-NH_2_ into AZM-SH was also confirmed by the latter’s ^13^C NMR spectrum, in DMSO-d6 (Figure S3c). ATR-FTIR spectra of both intermediate AZM-NH_2_ and final product AZM-SH also translate into the above-mentioned modifications of the original AZM (Figure S4). Assigned vibration nodes (Table S1) are in accordance with those described in the literature for acetazolamide and its derivatives, specifically, those concerning analogous groups, such as -SO_2_NH_2_, amide (-C=O, >N-H and C-N-C), and thiadiazolic ring [48,50,51]. The appearance of preferable asymmetrical vibrational modes, in both AZM and AZM-SH, and the presence of particularly broader signals at low frequencies demonstrate their similar appetency to form intermolecular hydrogen bonds and dimeric conformations [48,52]. Special attention is brought to the vibrations concerning the >NH (amide) and -SO_2_NH_2_ groups in the spectrum of AZM-SH, whose significant broadening supports the already hinted formation of dimers via hydrogen bonding (Figure 2).

The successful synthesis of the intermediate AZM-NH_2_ and the final product AZM-SH were also confirmed with electron spray ionization (ESI) mass spectrometry (MS) analysis (Figure S5). With a nominal mass and a monoisotopic mass of 268 Da and 267.97585 Da (calculated), respectively, AZM-SH-[M]-was detected in two ionic forms: [M+H]^+^ (268.9831 *m/z*) and [M]^+^ (266.9853 *m/z*).

### 2.2. Docking Modelling and In Vitro Inhibition Studies

The effect of AZM-SH as potential inhibitor of human CAs, specifically the hCA IX isoform, was initially predicted by modelling the docking mechanism of AZM-SH into hCA IX active site. The AZM-less chain A of the 3IAI PDB model [53] was used for the determination of the best docking result. The ligand–receptor interaction depicted in the experimentally obtained 3IAI PDB model was taken as a comparative standard interactive conformation between AZM and hCA IX.

**Figure 3:**
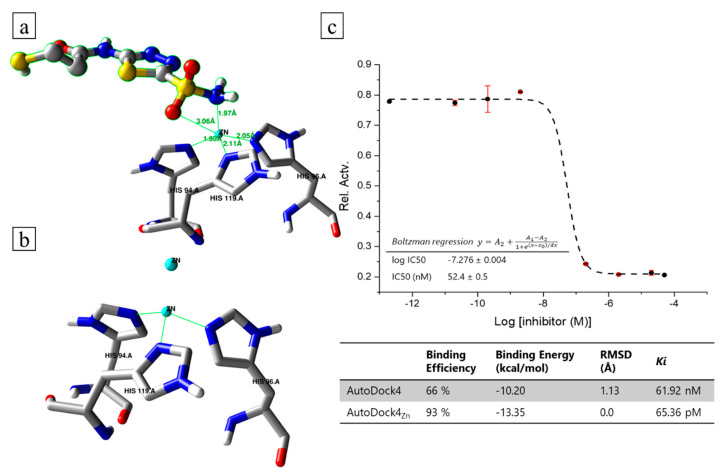
(**a**) Tetrahedral zinc geometry, with TZ pseudo-atom located at the unoccupied corner of the ideal coordination tetrahedral geometry. (**b**) AZM-SH zinc coordination via NA (nitrogen acceptor) atom-type interaction with the attractive component (TZ pseudo-atom) of the zinc ion. (**c**) CAs relative activity in the presence of AZM-SH and respective Boltzmann regression for the determination of its IC_50_. Docking performances for similar conformational modulations, using AutoDock4 and AutoDock4_Zn_ forcefields.

The AutoDock4_Zn_ forcefield was preferred over the traditional AutoDock4 forcefield, as its performance in properly reproducing zinc-coordination geometries has been proven to surpass the other’s predictions [54]. The chosen forcefield parameters consider the most common biological zinc coordination geometry, the tetrahedral one (Zn_3.1_ and Zn_4.0_), where AutoDock atom types NA (nitrogen HB acceptor), N (nitrogen non-HB acceptor), OA (oxygen HB acceptor), and SA (sulphur HB acceptor) and their interaction and positioning regarding the ZN-type atom play an important role in the proper docking of ligand molecules (Figure 3a). Moreover, the inclusion of a TZ pseudo-atom mediating the attractive component of the zinc ion allows a new directional tetrahedral potential for the NA atom type and more accurate docking results, with lower binding energies and RMSD values and higher binding efficiencies (Figure 3c).

As expected, the highest binding efficiency (93%) and the lowest binding energy (−13.89 kcal/mol), RMSD (0.0) and inhibition constant (Ki: 65.36 pM) were obtained for a conformation where the sulfonamide moiety formed a direct interaction (1.97 Å) with the Zn^2+^ ion in a tetrahedral orientation, via coordination by the N(13)-atom of the primary amine. Like the reported AZM-hCA IX interactive complex, the Zn^2+^ ion is also coordinated (3.06 Å) by one of the adjacent O-atoms of the sulfonamide moiety (Figure 3b). Likewise, the non-coordinating O-atom and the H(13)-atom of the amine, present in the sulfonamide moiety, were predicted to form hydrogen bonds with the GLU106 (2.72 Å) and THR199 (1.98 Å) residues of hCA IX, respectively (Figure S6a) similar to other reported results [26,55,56].

Hydrogen bonding was also calculated to occur between the hydrophilic = N(8)-, >N(6)H, =O(4), -S(1)H groups of the ligand, adjacent water molecules and the THR200, PRO201, and GLN67 GLN92 receptor residues, forming a continuous H-bond network facing the outer surface of hCA IX active site (Figure S6b,c). Accordingly, the alignment of the aliphatic C(3) and C(4) of the sulfanylpropanamide segment of AZM-SH with internal hydrophobic moieties of the LEU198, LEU141, VAL121, and VAL143 residues (Figure S6d) contributes to the overall stability of the obtained conformation.

A similar conformation was obtained using the standard AutoDock4 forcefield, and without the inclusion of the TZ pseudo-atom, but only for a lower binding efficiency and higher binding energies, RMSD value, and Ki (Figure 3c).

The preferable AutoDock4_Zn_ predicted ligand–receptor interactions and spatial arrangement (Figure S6) indicate that the chemical modification of the original AZM to include the sulfanylpropanamide segment, has little to no hampering impact on the inhibition role of the final AZM-SH ligand, making it a good potential inhibitor for the CRC-associated hCA IX isoform.

Experimental inhibition activity of the synthesised AZM-SH against CAs was assessed using an esterase-mediated assay and calculated after appropriate Boltzmann regression (Figure 3c). The obtained AZM-SH IC_50_ of 52.4 ± 0.5 nM (n = 3) is well within the same order of magnitude as that reported by the kit’s manufacturer for the standard AZM (IC_50_ = 16.3 ± 2.0 nM), and as other IC_50_ reported in the literature for other AZM derivatives [55,57]. The assay confirmed the final product’s efficient inhibition activity towards CAs, namely hCA IX, thus supporting its use as potential targeting agent in future anticancer therapies.

### 2.3. NANO3-SH Characterization

As demonstrated in a previous work of our group [34], the synthesis of NANO3-SH resulted in well-defined spherical, single core–shell particles with an average diameter of 60 nm and a shell porous matrix with pores of ca. 2.5–3.0 nm.

**Figure 4:**
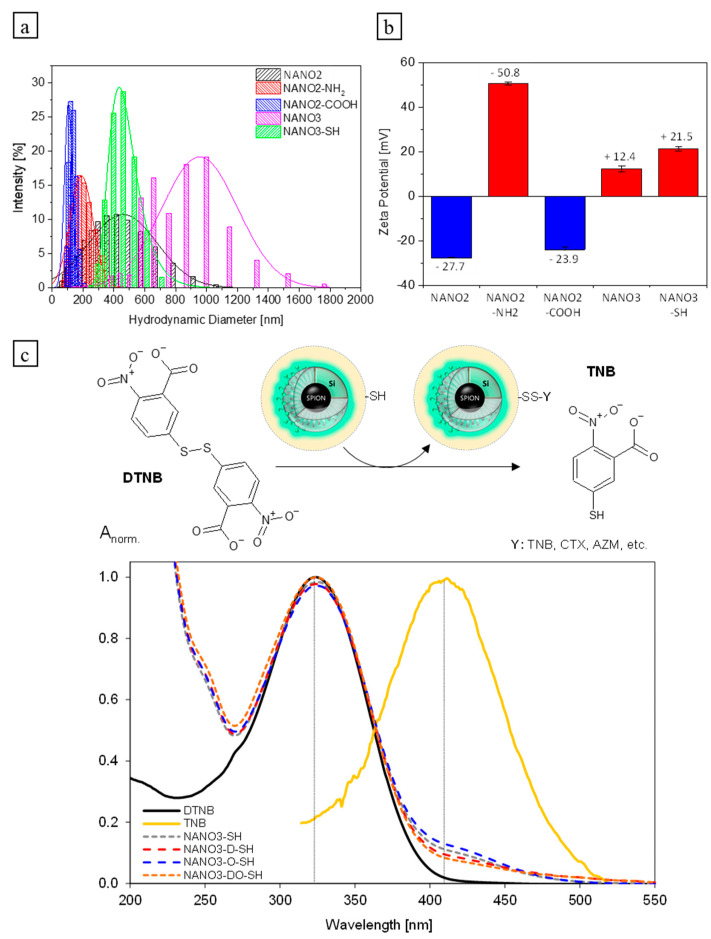
(**a**) DLS hydrodynamic diameter of NANO2 to NANO3-SH systems; (**b**) respective surface ζ; (**c**) molecular interaction of Ellman’s reagent with thiol moieties and corresponding spectroscopic response in obtained supernatants (i.e., absorbance band red shift to 412 nm) upon the formation of TNB-free species; NANO3-SH systems spectroscopic signature upon thiol determination and activation.

The assessment of NANO surface charges by DLS and zeta potential throughout the synthetic steps was an important tool to not only quickly confirm surface modifications but also evaluate NANO3’s polydispersity and stability in the selected medium. This factor has a high impact on the performance of the nanovectors by having an active role in drug-delivery and cell-uptake processes [58]. As depicted in Figure 4, the thiolation of NANO3 surface via EDC/NHS cross-linkage of SiQD available amines and TGAc carboxylic groups rendered the final NANO3-SH with increased stability in the physiological medium, translated by an increase in zeta potential (ζ) from +12.4 mV to +21.5 mV and a clear reduction in its hydrodynamic diameter and polydispersity. The more robust net positive charge after thiolation makes NANO3-SH particularly interesting for cellular therapeutic applications, namely CRC therapy, as positively charged particles have been recurrently reported to have preferential binding activity to cell membranes and increased uptake [58]. Moreover, enhanced cellular uptake has been reported for several thiol-containing nanoparticles, which covalently bind/interact with cell surface thiol groups, increasing 100-fold the membrane transfection efficiency and endocytosis [59].

The introduction of thiol terminal moieties to NANO3-SH systems sought also to create binding points for further biofunctionalization with anti-CA IX and anti-EGFR species. Consequently, it was not only important to assess the thiolation ratio but also necessary to activate these thiol moieties and catalyse further linkages. Ellman’s reagent (DTNB) has been repeatedly reported as a good visual and spectroscopical agent for the estimation of disulphide bonds and existing thiol moieties, owing to the surgency of a quantifiable absorbance band at 412 nm (translated by a yellow solution) [60,61,62] (Figure 4). Thiolation was applied to bare NANO3-SH (0.33 ug_SH_/mg_NANO_) and to its loaded counterparts, NANO3@D-SH (0.28 ug_SH_/mg_NANO_), NANO3@DO-SH (0.25 ug_SH_/mg_NANO_), and NANO3@O-SH (0.38 ug_SH_/mg_NANO_), rendering them with 0.25, 0.25, 0.19 and 0.29 nmol possible S-binding, active sites for further modification with thiol-containing biomolecules/small molecules such as the CTX-SH and AZM-SH synthesized herein.

A last assessment of the NANO3-SH physical stability was performed by exposing the nanosystem to simulated in-body conditions, this is, at 37 °C under continuous stirring in 0.01 M PBS at physiological pH, for one month. From the TEM micrographs (Figure S7), a total decomposition of the structured material is seen with all magnetic cores segregated from the present silica, and the latter spread through the whole field with no structured order.

### 2.4. Loading and Release Assays. Proof of Concept: Enteric Coatings and Gastric Simulated Release

The empty porous matrix of NANO3 was used to load both a model anticancer and an antibiotic, in single and dual formulations, to exploit existent synergetic effects between them such as improved therapeutic activity and release parameters, as previously demonstrated by our group [34]. The loading of both drugs was performed in an intermediate step of NANO3-SH synthesis, via electrostatic interactions with non-templated NANO2-COOH, before pores capping with SiQDs and thiolated species. Accessibility to pores is high at this stage, where surface moieties are yet malleable and do not produce a high steric impediment on drug migration. Moreover, the correct selection of loading media’s pH (pH 7.4) was important to maximize attractive interactions between the negatively charged -COOH surface moieties, pores, silanol groups, and both doxorubicin (DOX) (pKa-NH_2_ = 8.2, pKa(phenol) = 9.5) and ofloxacin (OFLO) (pKa-COOH = 6.1, pKa(piperizinyl ring) = 8.2) [63,64], which are partly positively charged at physiological pH (Figure S8a).

**Figure 5:**
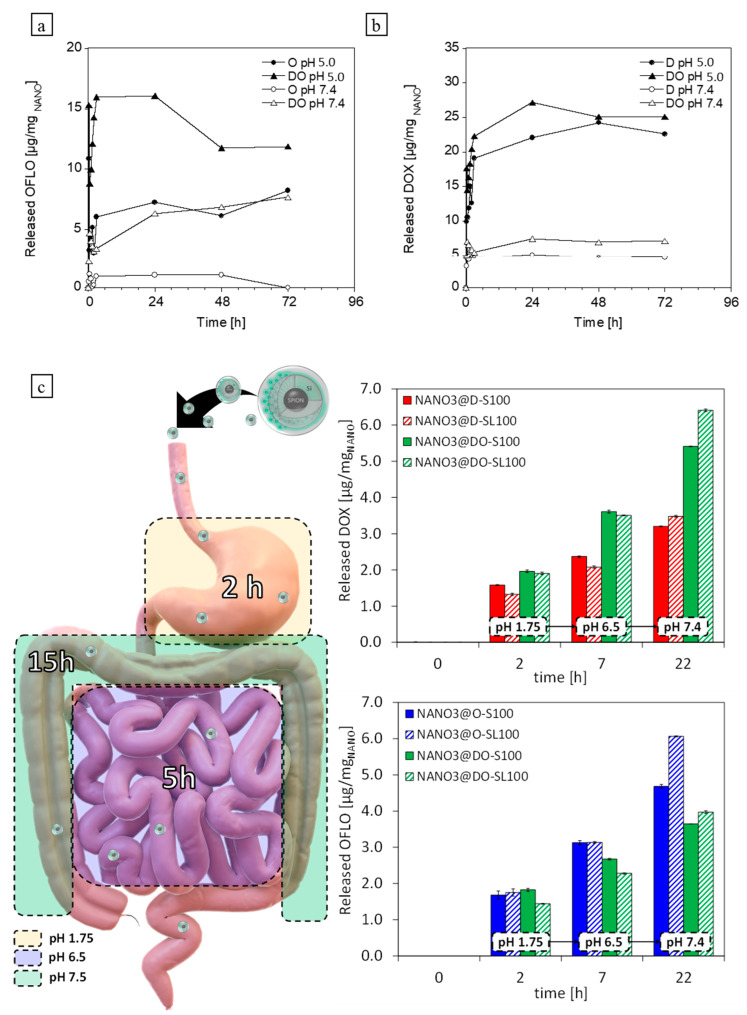
(**a**) Ofloxacin (OFLO) and (**b**) doxorubicin (DOX) pH-stimulated release profiles from NANO@O-SH, NANO@DO-SH and NANO@D-SH, at pH 7.4 and 5.0, with the latter significantly boosting DOX release; (**c**) simulated digestive track release from enteric-coated NANO3@drugs systems (n = 3), with medium pH and exposure time fitting the biological conditions of the selected sections of the digestive tract (yellow—stomach; blue—small intestine; green—colon).

DOX and OFLO were successfully loaded inside NANO2-COOH, and its drug content was evaluated by UV-Vis (λ_DOX_ = 480 nm; λ_OFLO_ = 330 nm) at each surface modification step, till the final product NANO3-SH, before its biofunctionalization (Figure S8b). The obtained loaded nanosystems and its corresponding drug content are NANO3@D-SH with 287.5 µg_DOX_/mg_NANO_, NANO3@DO-SH with 274.0 µg_DOX_/mg_NANO_ and 140 µg_OFLO_/mg_NANO_, and NANO3@O-SH with 182.8 µg_OFLO_/mg_NANO_. The high encapsulation capacities are in accordance with those reported in previous studies for similar mesoporous systems and selected drugs, with OFLO successfully replicating the results obtained in other works by our group [65,66,67,68]. Regarding NANO3@DO-SH formulation, the relatively reduced DOX solubility at the selected medium pH contributes to the preferential loading of DOX, in detriment to OFLO encapsulation. The pH-dependent release capacity of each loaded system was performed with their non-biofunctionalized versions (i.e., NANO@drugs-SH, drugs = D, O, DO), to simulate the prominent stressful conditions in colorectal cancer microenvironments where there is redox imbalance and high levels of GSH promote disulphide bonds breakage [69,70]. CTX-SH and AZM-SH disulphide bridges with NANO3-SH systems are mostly broken in such conditions, with NANO3@drug–SH systems being representative of the release interface during its therapeutic action, either inside or outside CRC cells. The simulation of cancer microenvironment and its role in drug delivery from NANO3@drug–SH nanosystems was achieved by studying drug release in both physiological (pH 7.4) and acidic (pH 5.0) conditions (Figure 5a,b) for 72 h. When compared with the results obtained in previous studies [34] for non-thiolated NANO3 systems, under the same conditions, one concludes that although the final functionalization with thiol moieties does not change the release tendencies for different pHs, it changes the total released quantities of drugs. This effect is particularly significant for total released OFLO from both NANO3@O-SH and NANO3@DO-SH, which see, respectively, a 10-fold and 3-fold reduction when compared to their non-thiolated counterparts. Notwithstanding, the presence of DOX in a combinatory formulation promotes, again, a higher release of OFLO (although lower than that of the aforementioned NANO3 systems). The slow release of OFLO from NANO3 systems is, however, an interesting result, as a more time-paced delivery of the antibiotic could promote an initial therapeutic burst of anticancer activity by DOX, followed by a later, long-term, and continuous antimicrobial action of OFLO.

Conversely, DOX release either remains almost invariable or is enhanced. While for NANO3@D-SH released DOX at pH = 7.4 is the same as that of NANO3@D, at acidic conditions, it sees a 2-fold increase. Regarding NANO3@DO-SH, no changes were seen when compared to its non-thiolated counterpart. Overall, the release of drugs, DOX in particular, is favoured in acidic microenvironments and in DOX:OFLO dual formulations.

As a proof-of-concept, NANO3@drugs-SH systems underwent an extra modification: a layered protective enteric coating with Eudragit^®^ polymers (PMMA-MMA copolymers). These enteric polymers are well-known protective excipients for active pharmaceutical compounds, capable of regulating their release at different conditions of interest, be they time-based, pH-based or temperature-based [41,71,72]. Of all available Eudragit^®^ polymers, E-S100 and E-L100 polymers were selected based on their renowned reputation for colon-targeted drug delivery [73,74] and their dissolution pH thresholds, with the former preferably dissolving at pH > 6.0 and the later at pH > 7.0. Their dissolution pH coincides with those of small intestine and colon sections (i.e., ascending, transverse, and descending), where a proper dissolution of their polymeric matrix promotes the release of encapsulated NANO3 systems and the effective/local delivery of their targeting and therapeutic agents to CRC cells.

Two distinct encapsulation approaches were performed to assess the effective delivery to the colon and understand the protective role of the enteric matrices in NANO3 systems delivery: (i) a single encapsulation with E-S100 (i.e., NANO3@drugs-S100) and (ii) a dual 1:1 encapsulation with E-S100 and E-L100 (i.e., NANO3@drugs-SL100). The successful coating of NANO3-SH and NANO3@drug–SH particles was followed by zeta potential after the addition of each enteric coating as a total inversion to negative surface charges was expected [72]. The total inversion of their zeta potential (in deionized water, pH ~6.5), from a net-positive thiolated system to a net-negative system with average ζ = −24.0 ± 1.7 mV, confirmed indeed the successful coating by E-S100. A secondary coating with E-L100 was again confirmed by zeta potential, with obtained NANO3-SH materials showing consistent negative zeta potentials.

A simulated digestive track delivery and colonic-targeted release were achieved by exposing coated NANO3 systems to different pHs and stirring times, in accordance with the conditions and passage times typical of each section. As perceived in Figure 5c, and in line with previously reported works on E-S100 and E-L100 [74,75], the overall release of drug-loaded nanoparticles in the simulated digestive fluids is higher at colonic pH than at small intestine and stomach pH. This demonstrates the capacity of the enteric coatings to protect the encapsulated NANO3@drugs-SH systems from external conditions, conditionate its release for the appropriate section of the digestive track, and increase its bioavailability.

Regarding single E-S100 and dual E-SL100 formulations, the results show that at a pH of 1.75 and 6.5, the dual enteric formulation shows an overall better capacity to control/retard the release by dispensing less NANO3@drugs-SH to the medium than E-S100 formulations at the same pHs. The early relaxation and dissolution of the E-S100 expedite NANO3@drugs-SH exposure to surrounding media and the release of the loaded drugs at an early stage. This tendency is delayed in the dual-enteric formulations, for which, at only a pH of 7.4 (i.e., in the colon), the external E-L100 coating is dissolved and a higher dispensing of nanoparticles to the surrounding media is permitted. The obtained results are in accordance with other reports that use the same model drugs [76,77] supporting the application of these enteric coatings in the oral administration of a sustained delivery to the colon.

### 2.5. Biofunctionalization

The biofunctionalization of the already activated NANO3-SH systems with the previously modified CTX-SH and the synthesized AZM-SH was achieved by promoting the formation of disulphide bonds between nanoparticles and biological species. Biofunctionalized nanoparticles were obtained in three distinct final formulations: (1) single functionalization with thiolated CTX-SH (NANO3-CTX and NANO3@drugs-CTX), (2) single functionalization with AZM-SH (NANO3-AZM and NANO3@drugs-AZM); and (3) dual functionalization with CTX-SH and AZM-SH (NANO3-CTX and NANO3@drugs-CTX). A summary of all final formulations is depicted in Table 1.

Surface-bound CTX content in NANO3 systems was assessed by forced reduction in cross-linking disulphide bonds and gel electrophoresis quantification (free CTX was used as standard) (Figure S9). Surface-bound AZM content was confirmed and determined by UV-vis spectroscopic analysis, at 412 nm, of the differential content of generated free TNB upon biofunctionalization of the activated (TNB containing) NANO3-SH/NANO3@drugs-SH, with AZM or CTX+AZM.

As shown in Table 1, a significant increase in the co-functionalization of CTX and AZM occurred in the dual-loaded NANO3 systems when compared to non-loaded NANO3 systems. This might have resulted from a higher availability of surface thiol groups, which are derived from an increased surface charge provided by the dual loading of OFLO and DOX (35 ± 0 mV for the dual loaded nanosystem, and 21.5 ± 1.0 mV for the non-loaded NANO3-SH). A similar trend is seen for NANO3@D-SH which has a ζ = 20 ± 0 mV, comparable to that of the empty NANO3-SH. This will result in fewer cross-linking phenomena between surface thiols and, subsequently, in a higher biofunctionalization rate for AZM and CTX. When regarding OFLO-containing NANO3 systems, there is also a variation in the surface charge (26.6 ± 0.9 mV); however, it is not as significant as that provided by the dual loading. This results in a more efficient biofunctionalization of the CTX and AZM molecules than that presented by the non-loaded system, but not as effective as that provided by the dual loaded system.

The occurring variations in surface charge are a response to the presence of one or two drugs and are influenced by the loading mechanisms of the drugs to the particles. These are modulated by their water solubility and electrostatic interactions between drugs and particles. The presence of ofloxacin always results in a higher surface charge, possibly due to the presence of a fluor atom in the molecule, which increases stereochemical impediment in the porous matrix, but is also modulated by ofloxacin solubility in water (1.4 mg/mL), which is higher than that presented by doxorubicin (0.3 mg/mL) [63,78]. With DOX presenting thus a lower water solubility, a loading into a more internal section of the mesoporous matrix is favoured, which is detrimental to a more external OFLO encapsulation.

### 2.6. Cytotoxicity, Antitumor Activity, Cellular Uptake, and Targetability

**Figure 6:**
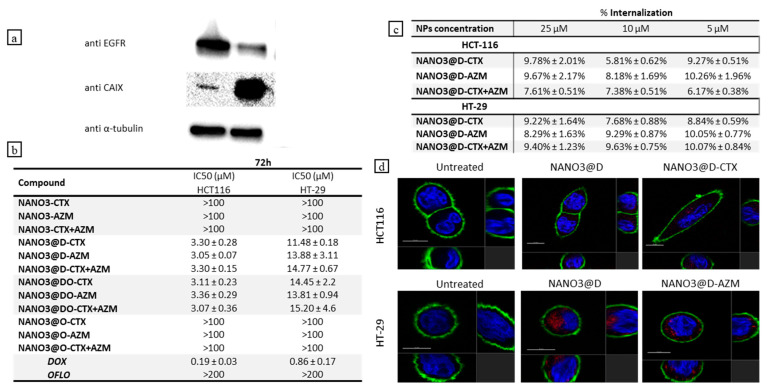
(**a**) Western blot for the overexpression levels of EGFR and CA IX in HCT-166 and HT-29 CRC cell lines; (**b**) IC_50_ values of biofunctionalized NANO3 and NANO3@drugs against HCT-116 and HT29 CRC cell lines, for 72 h. Concentrations are given as equivalent DOX concentrations (in µM); (**c**) internalization rates of different biofunctionalized NANO3@D systems, at different equivalent DOX concentrations (5, 10, and 25 µM) in HCT-116 and HT-29 CRC cell lines. Internalization rates given as the relative fluorescence of the samples against the fluorescence of the nanoparticles in the medium before treatment (excitation at 480 nm, emission collected at 550 nm); (**d**) LCSM images (images scale = 10 µm) of HCT-116 and HT-29 cell lines treated for 3 h with NANO3@D-CTX and NANO3@D-AZM, according to their specific overexpressed targets (untreated cells were used as negative controls).

The cytotoxicity of the biofunctionalized NANO3 systems was assessed against HCT-116 and HT-29 cell lines, as representative CRC models (Figure 6b). These cells were selected due to their hCA IX and EGFR overexpression, which was confirmed by Western blot, where high intensity bands are visible for anti-EGFR and anti-CA IX in the HCT-116 cell line and HT-29 cell line, respectively. These results indicate an EGFR overexpression in HCT-116 cells, whereas for the HT-29 cell line, there is an overexpression of the hCA IX receptor. The housekeeping α-tubulin protein was used as a control to normalize and ensure the presence of a similar cell concentration between the two lines (Figure 6a).

As per Figure 6b, the absence of the toxicity of the free OFLO supports the non-toxic role of the antibiotic in its nanometric formulations, which is further confirmed by the absence of toxicity from NANO3@O systems, regardless of the chosen CRC cell line. An equal lack of cytotoxicity was also observed for the empty NANO3 systems, which demonstrates the systems’ biocompatibility and possible usage in a human directed therapy. This is aligned with the cytotoxic profiles reported in the literature for similar composite nanomaterials, whose constituents have shown non-cytotoxicity to virtually null cytotoxicity [8,79,80].

The toxicity/antitumor activity of the prepared materials derives thus only from DOX present in their formulation, having a more accentuated action against the HCT-116 line than the HT-29 line. The obtained IC_50_ for the biofunctionalized NANO3 systems account for the total released DOX after 72 h and are in line with those obtained for the free drug. This demonstrates that the introduction of a protective nanometric material, capable of shielding DOX from harsh external conditions and in-body pharmacokinetics (i.e., absorption, distribution, metabolism, and excretion) [81,82,83], does not hinder the efficacy of the encapsulated drug and drug delivery.

To better understand the possible influence of the different biofunctionalizations (CTX, AZM and CTX+AZM) in the NANO3 systems, internalization assays were conducted for both cell lines. Exposure periods were predetermined at 3 h to evaluate the short time effects of each biofunctionalization in cellular uptake and at 72 h to assess the effects of a maximum cumulative uptake. After the predetermined exposure times, cells were stained and visualized by laser scanning confocal microscopy (LSCM). As depicted in Figure 6d, three distinct fluorescent signals were collected, referring to cellular membrane staining (green), nuclei position (blue), and DOX distribution (red). DOX was mostly localized in the cytoplasmic environment of both cell lines, confirming the successful internalization of NANO3@D-CTX and NANO3@D-AZM nanosystems.

A quantitative assessment of the uptake profiles was thus evaluated through cellular accumulation assays, in which cells underwent an exposure period for each nanoformulation of 3 h, similar to those presented in the aforementioned uptake assays with LSCM. Results depicted in Figure 6c show similar internalization percentages in regard to single and dual functionalized nanovectors, indicating a comparable targetability activity from the novel AZM derivative and the commercially available CTX, regardless of the type of biofunctionalization performed. Slight differences can be perceived when assessing internalization values such as a marginal increase in cellular uptake from NANO3@D-AZM (10.26 ± 1.96%; 10.05 ± 0.77%) in comparison to NANO3@D-CTX in the same respective internalization percentages (9.27 ± 0.51%; 8.84 ± 0.59%) for HCT-116 and HT-29 cell lines, respectively, in response to a concentration of 5 µM for both employed nanovectors. Although not excessively different, these alterations point to the possibility of a higher internalization degree in the presence of AZM derivatives as targeting agents.

## 3. Materials and Methods

### 3.1. Chemicals

N-hydroxysuccinimide (NHS. 98+%), ethylenediaminetetraacetic acid tetrasodium salt hydrate (EDTA. 98%), and tetraethyl orthosilicate (TEOS. ≥ 99.999% metals basis) were bought from AlfaAesar (Haverhill, MA, USA). N-3-dimethylaminopropyl-n-ethylcarbodiimide hydrochloride (EDC. ≥ 97%), acetone (≥99.5% GC), 4-morpholineethanesulfonic acid (MES. ≥ 99%). thioglycolic acid (TGAc. 98%), 5.5′-dithiobis(2-nitrobenzoic acid) (Ellman’s Reagent. DTNB. ≥ 98%), 2-2-iminothiolane hydrochloride (≥98% (TLC)). 5-acetamino-1.3.4-thiadiazole-2-sulfonamide (Acetazolamide. AZM. ≥ 99%), 3-mercaptopropionic acid (99%), anhydrous N.N-dimethylformamide (DMF. 99.8%), anhydrous tetrahydrofuran (THF. 99.9%), TRIS (hydroxymethyl)aminomethane hydrochloride (TRIS HCl), doxorubicin hydrochloride (DOX. 98.0–102.0% (HPLC)), DL-Dithiothreitol (DTT), β-mercaptoethanol (molecular biology grade), oxalyl chloride (>99%), and phosphate-buffered saline (PBS tablets) were purchased from Sigma Aldrich (St. Louis, MO, USA). Hydrochloric acid (HCl. 37%) and sodium hydroxide (NaOH) were bought at Scharlab. SL (Barcelona, Spain). Ofloxacin (OFLO. 98%) was bought from ThermoFisher (Waltham, MA, USA). Deuterated reagents as dimethyl sulfoxide-D_6_ (DMSO-d_6_). Acetone-D_6_ and acetonitrile-D_3_ (CD_3_CN) were purchased from Acros Organics (Geel, Belgium). Anti-EGFR Cetuximab (CTX 5.2 mg/mL. 100%) was acquired from Selleckchem (Houston, TX, USA). Eudragit-L100^®^ and Eudragit-S100^®^ were supplied by Evonik Operations GmbH (Essen, Germany). All reagents and solutions were used as acquired without any further purification.

### 3.2. Instrumentation

ATR-infrared spectra were collected in a Tensor 27 (Bruker, Billerica MA, USA) and ^1^H/^13^C nuclear magnetic resonance spectroscopy (NMR) performed in a Bruker FT-NMR Avance 400 (Ettlingen, Germany) spectrometer at 300 K from the Departamento de Química, Universidad Politécnica de Valencia. Mass spectroscopy analysis was performed by ESI+QTOF MS in a SCIEX 6600 plus TripleTOF of the SCSIE-UVEG of the University of Valencia. Melting point was determined in a Gallenkamp apparatus (England) from the Bioscope Group LAQV-NOVAFCT. Elemental analysis was performed by CHNS method in an Elemental analyzer Thermo Finnigan-CE Instruments Flash EA 1112 CHNS series from the Analysis Laboratory at LAQV-NOVAFCT. Ninety-six-well plate sampling analysis was performed in an EnSpire^TM^ multilabel reader (Perkin Elmer. Singapore Pte. Ltd., Singapore) from Departamento de Química, Universidad Politécnica de Valencia.

Temperature controlled incubations and release studies were performed in an HC24 PCMT Thermo-shaker (Grant Instruments, Cambridge, UK). DLS experiments were performed in a Malvern Nano ZS Zetasizer (633 nm laser diode). UV–vis absorption spectra were obtained in a Jasco V-650 spectrophotometer and fluorescence spectroscopy measurements in a HORIBA Scientific FLUOROMAX-4 spectrofluorometer from the Bioscope Group LAQV- FCT NOVA and a JASCO FP-8300 spectrophotometer from UPV.

Cell images were taken using a laser scanning confocal microscope (LSCM) SP5 (Leica), and cell viability was measured in a multilabel plate reader (Victor 3 PerkinElmer).

### 3.3. Synthesis and Characterization

#### 3.3.1. N-(5-Sulfamoyl-1,3,4-thiadiazol-2-yl)-3-sulfanylpropanamide (AZM-SH) Synthesis

The first step for the synthesis of the novel N-(5-sulfamoyl-1,3,4-thiadiazol-2-yl)-3-sulfanylpropanamide (**AZM-SH**) was the production of both starting materials: 5-amino-1,3,4-thiadiazole-2-sulfonamide (**AZM-NH_2_**) and 3-sulfanylpropanoyl chloride. A schematic overview of the complete synthetic route is depicted in Scheme 1.

**AZM-NH_2_** was produced following a procedure patented by Kandula [84]. Briefly, 1.5 g (6.75 mmol) of 5-acetamino-1,3,4-thiadiazole-2-sulfonamide (**AZM**) was thoroughly dissolved in a mixture of ethanol (10.0 mL) and HCl 37% (3.0 mL, 12M) and refluxed for 4.5 h. Refluxing was followed by cooling and solvent evaporation under vacuum. The resulting solid residue was redissolved in MilliQ H_2_O (7.5 mL) and neutralized to pH 7.0 with a 5 M aqueous solution of NaOH. Upon neutralization, a white precipitate was formed, which was collected by filtration and recrystallized in water overnight. **AZM-NH_2_** was then collected in the form of white crystals (yield of 40% wt.), ESI+QTOF MS: [C_2_H_4_N_4_O_2_S_2_+H]^+^ 180.9848 *m/z* (error—3.8 ppm). ^1^H NMR spectrum (DMSO-d6, 400 MHz) δ (ppm): 7.81 (s, 2H, H11-NH2), 8.05 (s, 2H, H1-NH2). ^13^C NMR spectrum (DMSO-d6, 400 MHz) δ (ppm): 158.61 (C2), 172.05 (C5). The product was also characterized by ATR-FTIR.

The chlorination of 3-mercaptopropionic acid, for 3-sulfanylpropanoyl chloride formation, was accomplished by a typical Vilsmeier mechanism, as detailed by Mohapatra et al. [85]. A solution of 3-mercaptopropionic acid (0.87 mL, 10.0 mmol), in DCM (5 mL), was firstly cooled to 0 °C, under N_2_ atmosphere. To this mix, catalytic amounts of anhydrous DMF were added, followed by a dropwise addition of oxalyl chloride (2.0 mL, 20 mmol). The resulting mixture was then slowly stirred for 4.5 h at room temperature. Solvents and excess oxalyl chloride were removed by evaporation under vacuum. The product, 3-sulfanylpropanoyl chloride, was obtained in the form of a dark-brown liquid and characterized by ^1^H-NMR and ^13^C-NMR.

For the production of the final product, N-(5-sulfamoyl-1,3,4-thiadiazol-2-yl)-3-sulfanylpropanamide, both starting materials were combined in an adapted protocol from those described by de Simone et al. [26] and Bolla and Nangia [86]. Ninety milligrams (0.5 mmol) of AZM-NH_2_ was dissolved in THF (5 mL) and mixed with 50 µL (1 mmol) of 3-sulfanylpropanoyl chloride, at room temperature and N_2_ atmosphere. After 12 h of continuous stirring, the solvent was evaporated under vacuum and the obtained green precipitate was left to dry for 48 h under vacuum. The dry pellet was then redissolved in MilliQ H_2_O, tuning from its original green colouration to a brownish-white colour and filtered under vacuum with five consecutive washes in water, dichloromethane, and diethyl ether.

The resulting product, N-(5-sulfamoyl-1,3,4-thiadiazol-2-yl)-3-sulfanylpropanamide (**AZM-SH**, FW = 268.34), was dried at 37 °C and collected in the form of brownish-white powder (70.4% wt. yield, mp 203.6 ± 1.0 °C), ESI+QTOF MS: [C_5_H_8_N_4_O_3_S_3_+H]^+^ 268.9831 *m/z* (error—2.5 ppm) and [C_5_H_8_N_4_O_3_S_3_-H]^+^ 266.9853 *m/z* (error—2.2 ppm). Elemental analysis of C_5_H_8_N_4_O_3_S_3_ was found (calculated): C 21.64% (22.38%), H 2.28% (3.00%); N 17.41% (20.88%), S 29.66% (35.85%).

^1^H NMR spectrum (CD_3_CN, 400 MHz) δ (ppm): 1.30 (s, 1H, H1 -SH) 2.70–2.97 (dt, 2H, H2 -CH2), 3.23–3.37 (dt, 2H, H3-CH_2_), 6.43 (d, 2H, H13-NH_2_, J = 33.8 Hz), 10.62 (br s, H, H6-NH-). ^13^C NMR spectrum (DMSO-d6, 400 MHz) δ (ppm): 24.04 (C1), 34.56 (C2), 161.29 (C3), 164.94 (C4), 170.78 (C5). The product was also characterized using ATR-FTIR.

#### 3.3.2. Synthesis of NANO3-SH

**Scheme 2:**
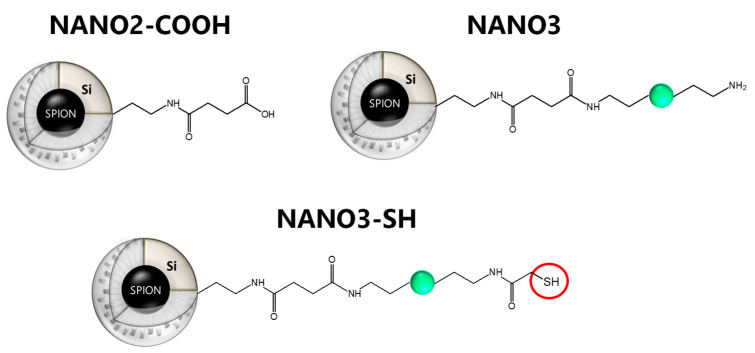
Structures of nanoparticles NANO2-COOH, NANO3, and NANO3-SH.

NANO3 nanoparticles previously synthesized in [34] were obtained using EDC/NHS cross-linkage of NANO2-COOH with the NH_2_ groups of the synthesized SiQDs, in a SiQD excess to avoid inter-NANO3 cross-linkage. The same strategy was used to thiolate NANO3 surface NH_2_ groups with thioglycolic acid (TGA), producing NANO3-SH (see Scheme 2). NANO3-SH activation of its surface thiol moieties was attained using Ellman’s reagent (DTNB), whose colourimetric change from transparent to yellow (412 nm) allows for a simultaneous estimation of the present -SH groups and degree of activation. All materials, at each step, were thoroughly characterized by TEM, DLS, and absorption/fluorescence spectroscopy.

### 3.4. Modelling Studies and Molecular Docking Simulations

Docking simulations were accomplished in AutoDock4, using the hCA IX crystal structure and active site, from the 3IAI protein complex, available at Protein Data Bank [53]. Docking parameters were defined according to AutoDock4_Zn_ forcefield, adapted to the docking of small molecules to zinc metalloproteins [54]. Ligand (AZM-SH) and receptor (hCA IX) were prepared separately. Ligand coordinates were processed with Openbabel [87] software to add hydrogen at pH 7.0. AutoDockTools v.1.5.6 [88] script *prepare_ligand4.py* with default settings was used to add Gasteiger–Marsili charges, merge non-polar hydrogens, and assign atom types. The number of torsions was set to 7 to include its rotatable amide bond. The receptor structure was prepared using AutoDockTools script *prepare_receptor4.py* with default settings, except the removal of water molecules, to assign Gasteiger–Marsili charges, merge non-polar hydrogens, add polar hydrogens, and assign atom types. The charge of the zinc ion was left to its default value (0.0) when using the Autodock4_Zn_ forcefield. The TZ pseudo-atom responsible for the attractive interaction in the tetrahedral coordination arrangement of zinc was added to the receptor molecule and to the standard forcefield table (Figure S6). Docking search parameters followed a Genetic algorithm, with 50 runs and a population size of 300. Three-dimensional representations of AZM, hCA IX, and docked complexes were performed with UCSF Chimera [89]. Modulations with the normal AutoDock4 forcefield, without the presence of the TZ pseudo-atom, were also run as comparison.

Best-fitting models were chosen for predictions with the lowest inhibition constant (Ki), the lowest binding energy and RSMD (<2.0 Å), and the highest binding efficiency.

### 3.5. CA In Vitro Inhibition Assay

Experimental in vitro AZM-SH inhibition activity was assessed using the CA Inhibitor Screening Kit (Colourimetric), by BioVision Inc., following the manufacturer’s instructions. The kit uses the esterase activity of an active CA on an ester substrate, producing a chromogenic product easily quantified by absorbance, at 405 nm. In the presence of a CA specific inhibitor, enzymatic activity decreases, accompanied by a reduction in the product’s absorbance. AZM-SH inhibitor 2 mM stock solution was prepared in 0.01 M PBS pH 7.4, and dilutions up to 2 pM were performed in the same buffer. The assay was prepared in a 96-well plate for AZM-SH concentrations of 2 mM, 1 mM, 0.5 mM, 0.2 mM, 20 µM, 2 µM, 20 nM, 2 nM, 0.2 nM, and 2 pM, and the absorbance was collected in a microplate reader at 405 nm, over the course of 60 min for 30 time points. Inhibition constant (Ki) was obtained as in vitro IC_50_ and compared to that of the standard AZM.

### 3.6. NANO3 Biofunctionalization with CTX-SH and AZM-SH

For each of the four 5 mg NANO3-SH samples, 400 µL of a cetuximab (CTX) 1 mg/mL stock solution in 0.01 M PBS pH 7.4 was mixed with 8 µL of a 1.93 mg/mL solution of 2-iminothiolane hydrochloride in 0.01 M PBS (pH 8.5), with 0.1 mM EDTA, for 1 h at 4 °C (in the dark). Each batch of the resulting thiolated cetuximab (CTX-SH) was 4 times centrifuged in a 10K MWCO Vivacon^®^ membrane at 13.500 rpm and 10 °C for 5 min. The CTX-SH was solvent-exchanged into a final volume of 100 µL of 0.01 M HEPES, with 0.1 mM EDTA ([CTX-SH]_final_ = 4.0 µg/µL).

For the biofunctionalization of the already activated NANO3-SH, 5 mg batches were used for each type of modification (i.e., single or dual modification). The 4 µg/µL (27.4 mM) CTX-SH solutions, in 0.01 M HEPES buffer, and a 1 mg/mL (3.73 mM) solution of AZM-SH, in 0.1 M Tris-HCl with 0.1 mM EDTA, were used as stock solutions. Briefly, 5 mg of NANO3 was resuspended in 0.1 M Tris-HCl (pH 8.05) to a total volume of 1.7 mL and mixed for 3 h at 4 °C (in the dark) with (1) 25 µL of CTX-SH stock solution; (2) 53 µL of AZM-SH stock solution; or (3) 25 µL and 53 µL of CTX-SH and AZM-SH stock solution. All samples were recovered by centrifugation, washed twice with 0.01 M PBS (V = 1 mL, pH 7.4), dried under vacuum, and sterilized by UV for 30 min (253.7 nm, 200 V, 50 Hz).

### 3.7. Drug Loading and Release Assays

Ofloxacin (OFLO) and doxorubicin (DOX) were used as antimicrobial and anti-tumoral model drugs and were loaded either alone or conjugated in a 1:1 wt. ratio. A 2 mg/mL doxorubicin stock solution was prepared in milliQ H_2_O and a 2 mg/mL ofloxacin stock solution in 0.01 M PBS (pH 7.0). Loadings were performed in batches of 20 mg of template-free NANO2-COOH. Briefly, 20 mg of NANO2 were resuspended in 3.2 mL of the corresponding drug stock solution and diluted in the same volume of the complementary solvent (miliQ H_2_O for ofloxacin and 0.01 M PBS for doxorubicin). Co-loading of OFLO and DOX was performed similarly, with 20 mg of NANO2-COOH being resuspended in 3.2 mL of OFLO and 3.2 mL of DOX stock solutions.

Each sample was collected by centrifugation (10,000 rpm for 10 min) and washed 4 times with 1 mL of PBS 0.01 M (pH 7.0). All supernatants were isolated and used to estimate, using a mass balance, the loading capacity of NANO2-COOH for each drug, via UV-vis spectroscopy at 330 (OFLO) and 480 (DOX) nm. The loaded nanoparticles were then submitted to the same surface modifications described above, yielding NANO3@D, NANO3@O, and NANO3@DO, and their DOX and OFLO content was assessed by the same procedure. Each product was further modified with CTX-SH and AZM-SH, accordingly.

pH-dependent release studies were conducted in PBS 0.01 M, with pH of 7.4, and pH 5.0, at 37 °C. Briefly, 1 mg of each loaded NANO3 was resuspended in 2 mL of buffer solution and shaken for up to 72 h. After centrifugation (12,000 rpm for 5 min), the released drug content in the supernatant was measured for the 5, 15, 30, 45, 60, 90 min, and 2, 3, 4, 24, 72 h time points, by fluorescence: OFLO at pH 7.4 (λ_exc_ = 300 nm, λ_exc_ = 460 nm), OFLO at pH 5.0 (λ_exc_ = 300 nm, λ_exc_ = 490 nm) and DOX (λ_exc_ = 480 nm, λ_exc_ = 560 nm).

### 3.8. Enteric Coatings and Simulated Digestive Track Release

The enteric coating of the final formulations was achieved using Eudragit^®^ polymers, Eudragit^®^ L100 (E-L100) and Eudragit^®^ S100 (E-S100), as model enteric coatings. Two types of coatings were prepared, one with only E-S100 and another with E-S100 followed by E-L100. Then, 0.2% *w/v* stock solutions of each polymer were prepared in ethanol. Briefly, 1 mg of each nanoparticle formulation was resuspended 1 mL of milliQ H_2_O and mixed with 250 µL of E-S100 stock solution overnight, at room temperature (in the dark). With all ethanol evaporated, all NANO3-S100 formulations were recovered by centrifugation (10,000 rpm, 15 min) and kept away from humidity in a desiccator. For further modification with E-L100, the selected products were treated with the same approach, but this time using E-L100 stock solution, yielding NANO3-SL100 formulations. All surface modifications were followed by DLS/Zeta characterization.

For a simulated gastric release, all formulations were suspended in the appropriate medium under continuous stirring at 37 °C, for the respective passage time of the simulated gastric section. All samples were collected (11,000 rpm, 3 min) after the appointed time, and the release amount of drugs was spectroscopically quantified. Firstly, the prepared formulations (1 mg) were resuspended in 2 mL of 0.025 M HCl (pH 1.75) for 2 h to simulate stomach conditions, followed by solvent exchange into another 2 mL of 0.01 M PBS (pH 6.5) for 5 h to simulate small intestine conditions and, lastly, by another solvent exchange into 2 mL of 0.01 M PBS (pH 7.0) for 15 h to simulate colonic conditions. Note that in this last part, the samples were quantified after 5 h and 15 h of incubation to assess the release of the drugs at ascending colon conditions and later at the end of the colon/rectum, respectively.

### 3.9. CA-IX and EGFR Expression in CRC Cell Lines

Human colorectal carcinoma cells (HCT 116) and human colorectal adenocarcinoma cells (HT-29) were obtained from American Type Culture Collection (ATCC, Manassas, VA, USA). HCT116 and HT-29 cells were routinely cultured in Dulbecco’s Modified Eagle Medium (DMEM, Invitrogen) containing 10% heat-inactivated foetal bovine serum and 1% antibiotic–antimycotic solution (Gibco) at 37 °C in a humidified 10% CO_2_ atmosphere.

HCT-116 and HT-29 cells in exponential growth were collected. 10^6^ cells of each cell line were pelleted, washed twice with PBS, and lysed using M-PER Extraction Reagent (Thermo Scientific). The total protein content was determined via a BCA protein assay (Thermo Scientific). Proteins from extracts from both cell lines were separated by size using SDS-PAGE and transferred into a PVDF membrane for Western Blotting. Epidermal Growth Factor Receptor (EGFR) and Carbonic Anhydrase IX (CA IX) levels were quantified after immunoblotting with anti-EGFR (Sc-03, Santa Cruz Biotechnology) and anti-CAIX (ref. 10107-R003, Sino Biological), respectively.

### 3.10. Cytotoxicity and Cellular Uptake

The cytotoxicity of biofunctionalized NANO3-SH systems (i.e., NANO3-CTX, NANO3-AZM, and NANO3-CTX+AZM) and their loaded counterparts (with DOX and OLFO) was tested in HT-29 and HCT-116 human CRC cell lines using the PrestoBlue assay. Cells were seeded into a 96-well plate at a cell density of 3.0 × 10^3^ cells/well and incubated for 24 h before nanoparticles were added at a concentration from 1 to 300 μg/mL. After 72 h, 20 μL of prestoBlue reagent was added to each well, and the plates were incubated for 4 h at 37 °C. The effect on cell viability was measured using a multilabel plate reader (Victor 3 PerkinElmer). Its fluorescence was quantified at λ = 615 nm (after excitation at λ = 531 nm), and its absorbance was monitored at λ = 570 nm. Cell cytotoxicity was evaluated in terms of cell growth inhibition in treated cultures and expressed as a percentage of the control conditions. Each experiment was repeated three times, and each concentration was tested in triplicate.

For cellular accumulation determination, HT-29 and HCT-116 cells on exponential growth were plated in 24-well plates at a density of 20,000 cells per well. Cells with exponential growth were plated 24-well plates, at a density of 20,000 cells per dish. Cells were grown overnight and treated with 5, 10, and 25 μg/mL of each nanoparticle for 3 h (untreated cells were used as negative controls). After treatment, cells were washed twice with PBS, detached with trypsin, resuspended, and lysed with PBS buffer with 1% of SDS. Fluorescence from lysates was measured in a Cary Eclipse fluorometer (samples were excited at 480 nm, and emission light was collected at 554 nm). The relative fluorescence of the samples was compared to the fluorescence of the nanoparticles in the medium before the treatment.

Cellular uptake experiments were also performed on HT-29 and HCT-116 cell lines. Cells with exponential growth were plated in MatTek^®^ 35 mm dishes, at a density of 40,000 cells per dish. Cells were grown overnight and treated with 25 μg/mL of NANO3@D-CTX, NANO3@D-AZM, and NANO3@D-CTX+AZM for 3 h (untreated cells were used as negative controls). After treatment, cells were counter-stained with Hoechst and CellMask and observed in a Confocal Microscope Olympus FV1000.

## 4. Conclusions

In light of the current need for new non-invasive and targeting therapies against CRC, herein, we detail the successful synthesis of a new acetazolamide derivative (AZM-SH), with anti-hCA IX inhibitory activity, dual-cargo, and dual-targeting magneto fluorescent nanosystem (NANO3) as a possible vehicle for a new orally administered anti-CRC therapy. Beyond their already confirmed antimicrobial activity, through the inclusion of an OFLO-containing formulation and hyperthermia properties, the NANO3 systems showed consistent and competitive anti-tumoral activity against both tested CRC cell lines. The inclusion of CTX and AZM derivatives as targeting agents at the surface of NANO3 showed benefits for their uptake in HCT-116 and HT-29 cell lines, particularly those functionalized with AZM-SH. Moreover, an oral delivery approach was confirmed through a proof-of-concept modification of the drug-loaded nanoparticles, with the inclusion of a combinatory enteric coating of E-S100 and E-L100 successfully delaying the release in simulated gastric and small-intestine conditions and favouring it in colonic conditions.

## Figures and Tables

**Table 1:** Summary of all final NANO3 formulations, with drug and targeting species content.

		Drug Loading		Biofunctionalization			
Sample	NANO3 (mg)	DOX (mg/g_NANO_)	OFLOX (mg/g_NANO)_	CTX (mg/g_NANO_)	AZM-SH (mg/g_NANO_)	CTX (#/NANO)	AZM-SH (#/NANO)
**NANO3-CTX**	5.0	-	-	13.65	-	5.54	-
**NANO3-AZM**	5.0	-	-	-	0.27	-	58.91
**NANO3-CTX+AZM**	5.0	-	-	5.62	0.33	2.28	72.39
**NANO3@D-CTX**	5.0	278.5	-	14.12	-	5.73	-
**NANO3@D-AZM**	5.0	278.5	-	-	0.32	-	69.85
**NANO3@D-CTX+AZM**	5.0	278.5	-	7.05	0.22	2.86	47.64
**NANO3@DO-CTX**	5.0	274.0	140.0	8.38	-	3.40	-
**NANO3@DO-AZM**	5.0	274.0	140.0	-	0.48	-	106.87
**NANO3@DO-CTX+AZM**	5.0	274.0	140.0	11.18	0.61	4.54	133.80
**NANO3@O-CTX**	5.0	-	182.8	9.76	-	3.96	-
**NANO3@O-AZM**	5.0	-	182.8	-	0.42	-	93.41
**NANO3@O-CTX+AZM**	5.0	-	182.8	6.13	0.35	2.49	77.97

All samples were resuspended in 1 mL PBS 0.01 M (pH 7.4) and then dried under vacuum. #/NANO is equivalent to the number of molecules per nanoparticle.

## Data Availability

The data presented in this study are available online within this article or in the Supplementary Materials.

## Supplementary Materials

The supporting information can be downloaded at https://www.mdpi.com/article/10.3390/ijms24076612/s1.
