# Supplementary material for: Development of New Targeted Nanotherapy Combined with Magneto-Fluorescent Nanoparticles against Colorectal Cancer
Source: Int J Mol Sci. 2023 Apr 1;24(7):6612. doi: 10.3390/ijms24076612 (PMC10095016; doi:10.3390/ijms24076612)
Supplement: Supplementary file 1 [file ijms-24-06612-s001.zip › ijms-2264800-supplementary.pdf]

Supplementary Material

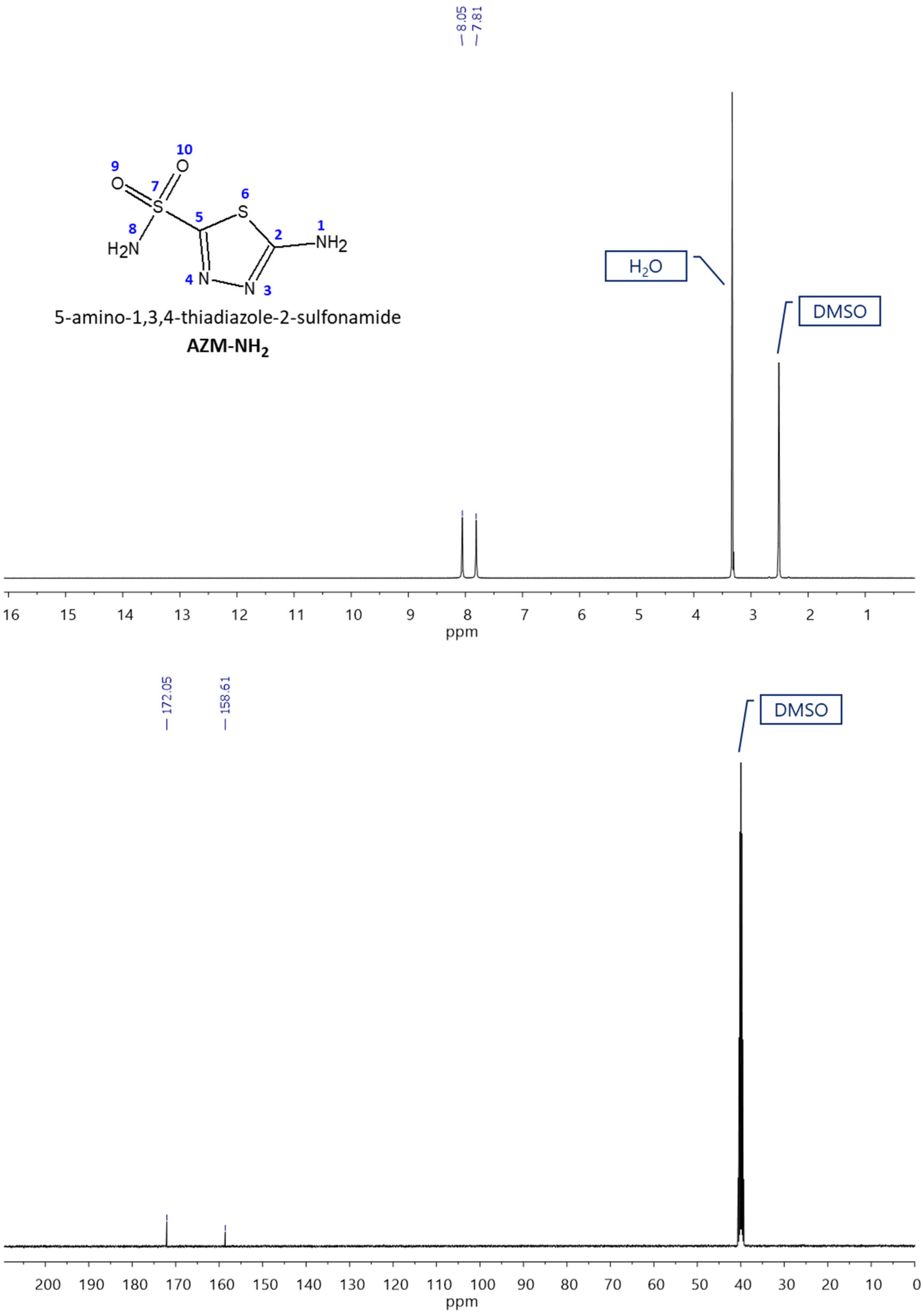

Figure S1. ***(top)*** ^{1}H NMR spectrum of AZM-NH_2: (DMSO-d_6, 400 MHz) δ (ppm): 7.81 (s, 2H, H8 -NH_2), 8.05 (s, 2H, H1 -NH_2); spectral contaminants: 3.33 (s, 2H, H_2O) and 2.51 (t, 6H, DMSO). ***(bottom)*** ^{13}C NMR of spectrum AZM-NH_2: (DMSO-d6, 400 MHz) δ (ppm): 158.61 (C2), 172.05 (C5); spectral contaminants: 39.35-40.61 (m, DMSO).

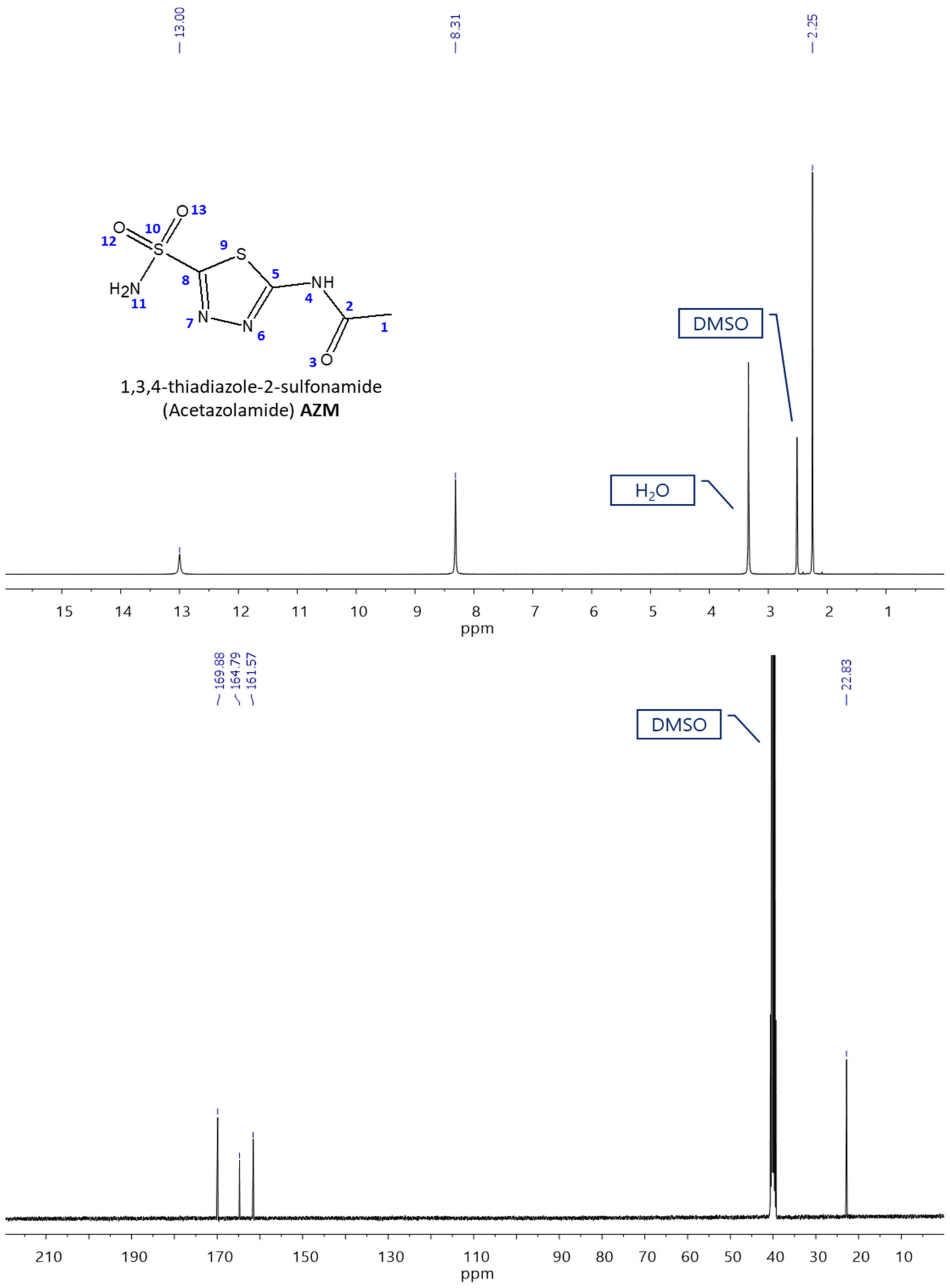

Figure S2. (*top*) ^{1}H NMR spectrum of AZM: (DMSO-d_6, 400 MHz) δ (ppm): 2.25 (s, 3H, H1 -CH_3), 8.31 (s, 2H, H11 -NH_2) and 13.00 (s, 1H, H4 -NH); spectral contaminants: 3.34 (s, 2H, H2O) and 2.51 (t, 6H, DMSO).

(bottom) ^{13}C NMR of spectrum AZM: (DMSO-d_6, 400 MHz) δ (ppm): 22.83 (C1), 161.57 (C2), 164.79 (C5) and 169.88 (C8); spectral contaminants: 39.37-40.62 (m, DMSO).

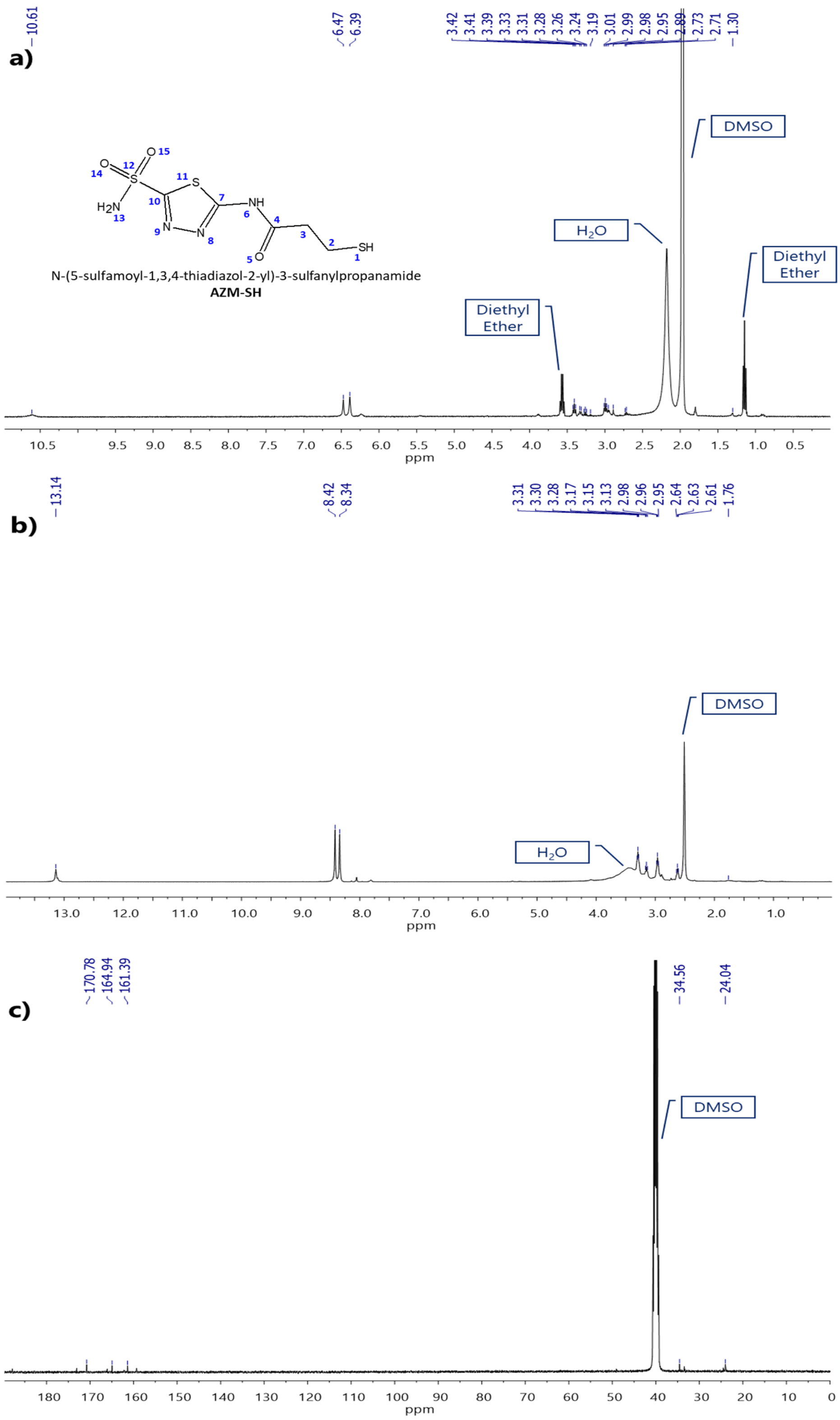

Figure S3. **(a)** 1H NMR spectrum of AZM-SH (CD_3CN, 400 MHz), spectral contaminants: 3.55 (q, 2H, $-CH_2$, diethyl ether) 2.22 (s, 2H, H_2O), 1.97 (s, 3H, CH_3CN), 1.15 (t, 3H, $-CH_3$, diethyl ether). **(b)** ^1H NMR spectrum of AZM-SH (DMSO-d_6, 400 MHz), spectral contaminants: 3.33 (s, 2H, H_2O) and 2.51 (t, 6H, DMSO). (c) ^{13}C NMR of spectrum AZM-SH (DMSO-d_6, 400 MHz), spectral contaminants: 39.35-40.61 (m, DMSO).

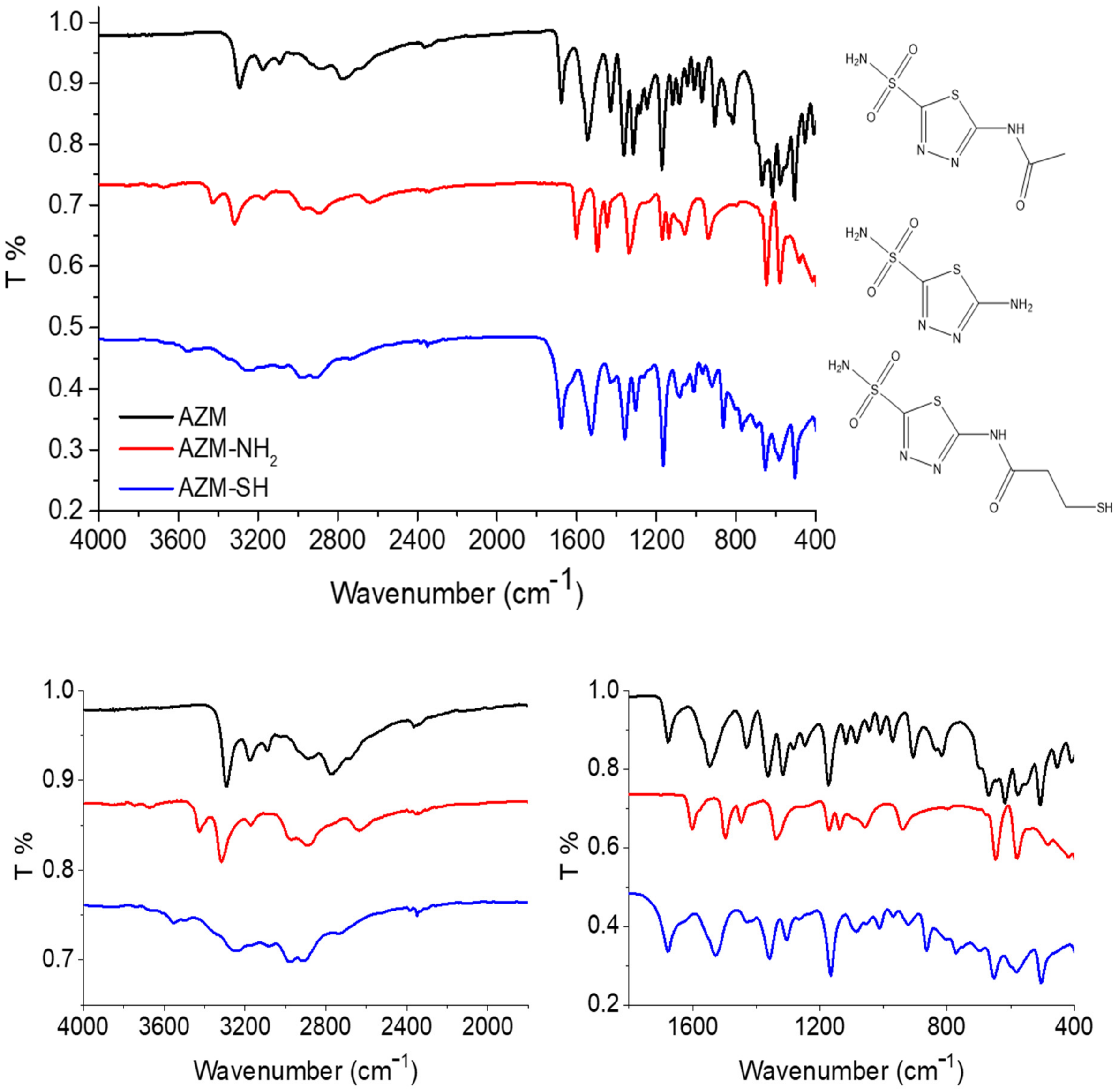

Figure S4. ***(top)*** Complete ATR-FTIR spectra for AZM. AZ-NH_2 and AZM-SH. ***(bottom)*** Close-up to the same spectra for the selected wavenumbers ranges.

Table S1. Assignment of all collected FT-IR peaks, for AZM, $AZM-NH_2$ and AZM-SH.

**AZM**	**$AZM-NH_2$**	**AZM-SH**	
FT-IR wavenumber (cm^{-1})			Assignment
-	3676	3676	ν_1
-	-	3549	ν_2
-	-	3491	ν_{as} N-H
-	3425 *str*	-	$R-NH_2$ *stretch*
-	-	3375 *brd sh*	ν_{as} $RSO_2N-H/-NH_2$
-	3317 *str*	-	$R-NH_2$ *stretch*
3294 *str*		3248 *str*	ν_{as} $RSO_2N-H/-NH_2$
3175 *med*	3170 *wk*	3170 *brd*	ν_s $RSO_2N-H/-NH_2$
3090	-	3086 *brd*	ν N-H (RCONH)
-	2974	2974	ν_6
2889	2889	2889	ν_7
2773	-	2723	ν ring-NH_2
2685	2638	-	ν_9
-	-	2515 *wk*	ν SH
2361	2341	2345	ν_{10}
1678 *str*	-	1678 *str*	C=O *stretch*<br>ν CO-NH-R
	1601 *str*		ν ring-NH_2
1543	-	1528	ν CO-NH-R<br>ν_{as} ring C=N
-	1497	-	ν azole ring
1431	-	1427	ν_s ring C=N<br>ν n-propyl/ethyl
-	1447	-	ν $R-SO_2-NH_2$
1362	-	1362	ν_{as} SO_2
-	1335	-	ν ring-NH_2
1311	-	1304	ν_{as} C-N-C
1284	-	1265	ν C-N ring
1246	1246	-	ν_{12}
1173	1173	1162	ν_s SO_2
1119	1138	-	ν_{13}
1084	-	1084 *med*	ν n-propyl/ethyl
1045	1057	1057	ν_{14}
1011	-	1011	ν_{15}
972	-	972 *wk*	ν n-propyl/ethyl<br>ν N-N
906	-	923	ν S-N<br>ν n-propyl/ethyl
-	-	864	ν n-propyl
837	-	-	ν ethyl
818	-	-	ν ethyl
-	-	802 *wk*	ν SH
-	-	771 *med*	ν_1 n-propyl
702	-	702	ν_{as} C-S ring
671	-	-	ν_{20}
-	648	652	ν_{21}
617	-	-	ν_{22}
579	579	582	δ $R-SO_2-NH_2$
555	-	-	ν_{23}
505	-	505	ν_{24}
455	482	-	ν_{25}
409	420	-	ν_{26}

wk, weak; *med*, medium; *str*, strong; *sh*, shoulder
s, symmetric; *as*, asymmetric

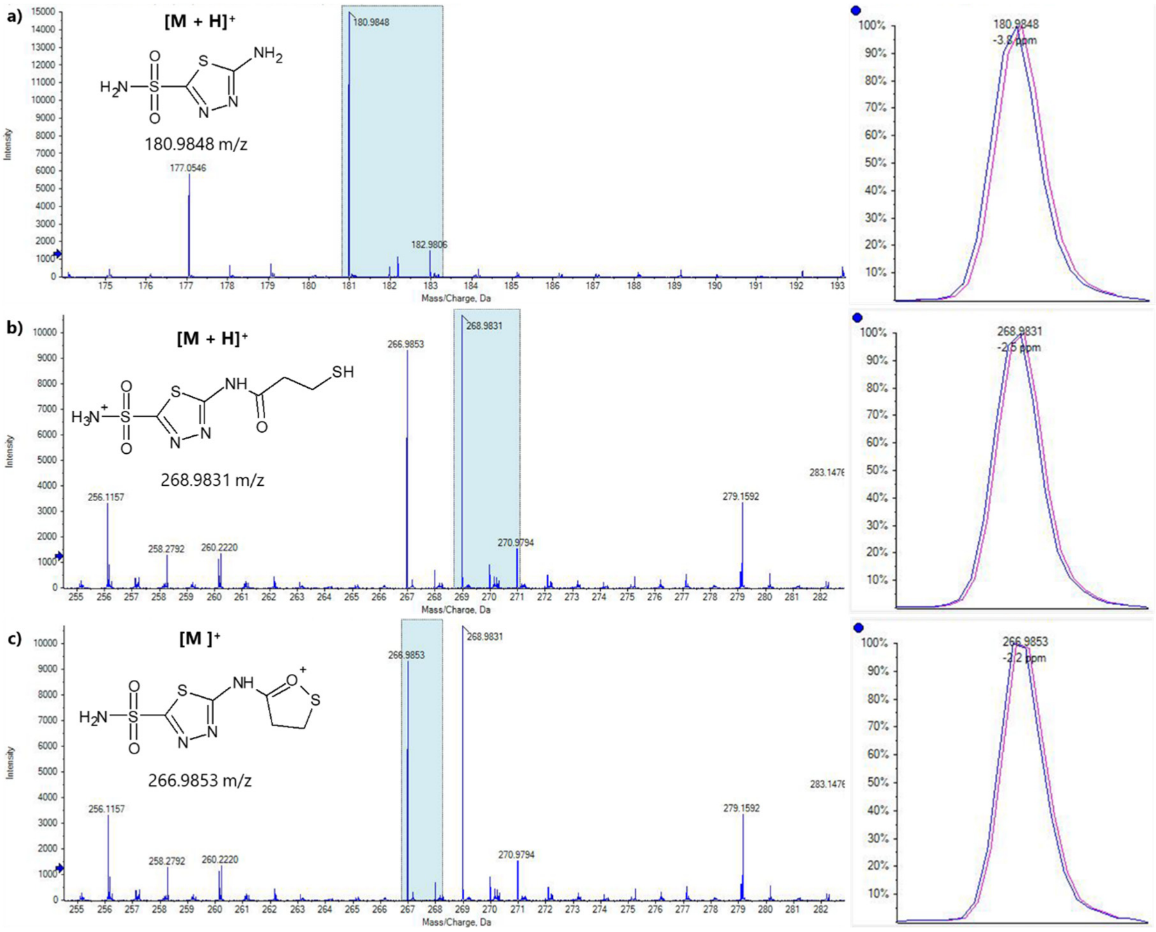

Figure S5. ESI+QTOF MS spectra of (a) AZM-NH_2. (b) AZM-SH $[M+H]^+$ ion and (c) AZM-SH $[M]^+$ ion.

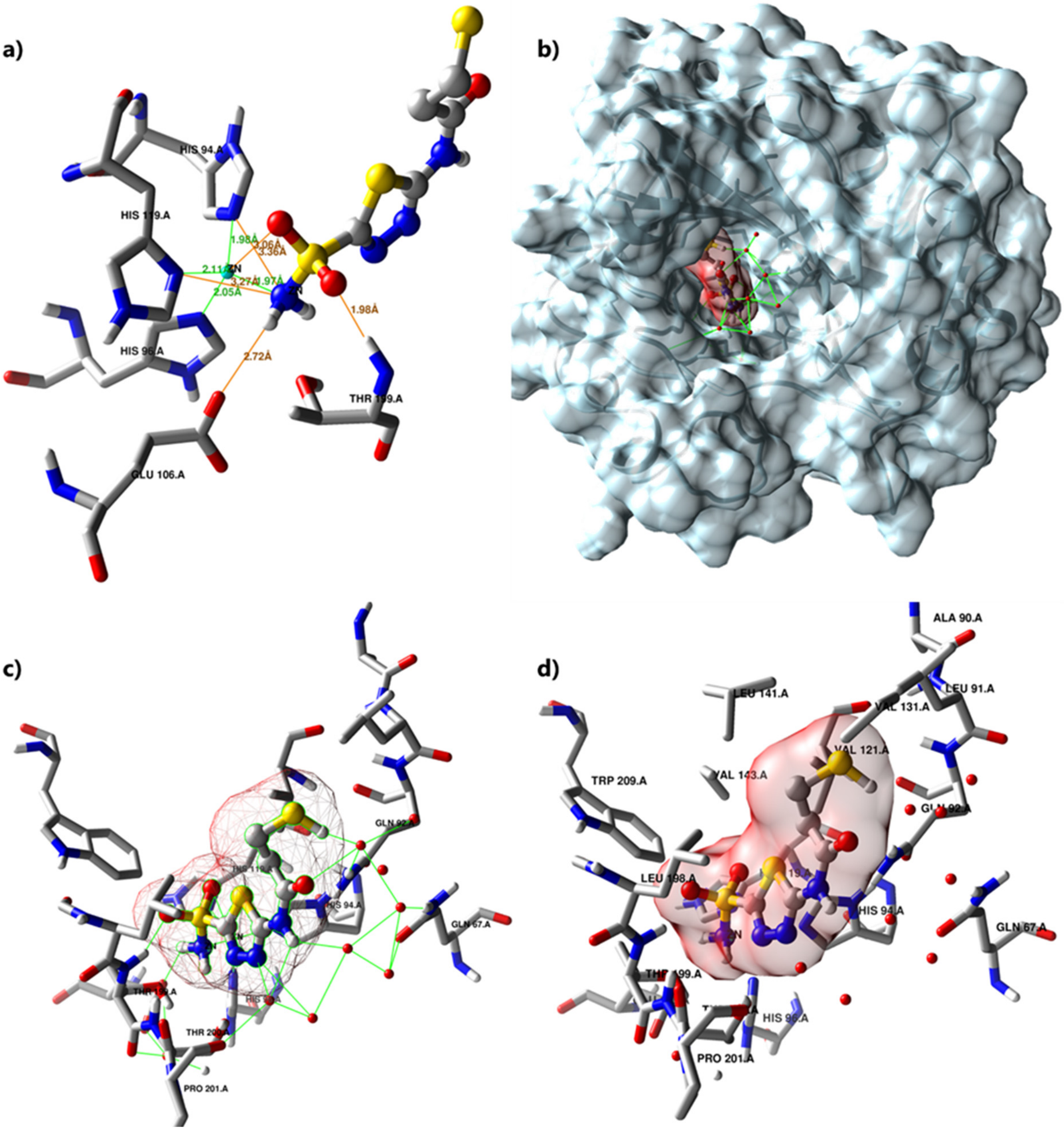

Figure S6. (a) Predicted zinc coordination interactions and hydrogen bonds for the AZM-SH sulfonamide moiety, with Zn^{2+} ion and GLU106 and THR199 CA IX residues, respectively. **(b)** Spatial surface representation of AZM-SH binding to CA IX active site. **(c)** Hydrogen bonds network between AZM-SH thiadiazole N(8)-atom, sulfanylpropanamide N(6), O(5) and S(1)H moieties, and water molecules. **(d)** Hydrophobic alignment of the aliphatic AZM-SH sulfanylpropanamide carbons and the hydrophobic residues of CA IX.

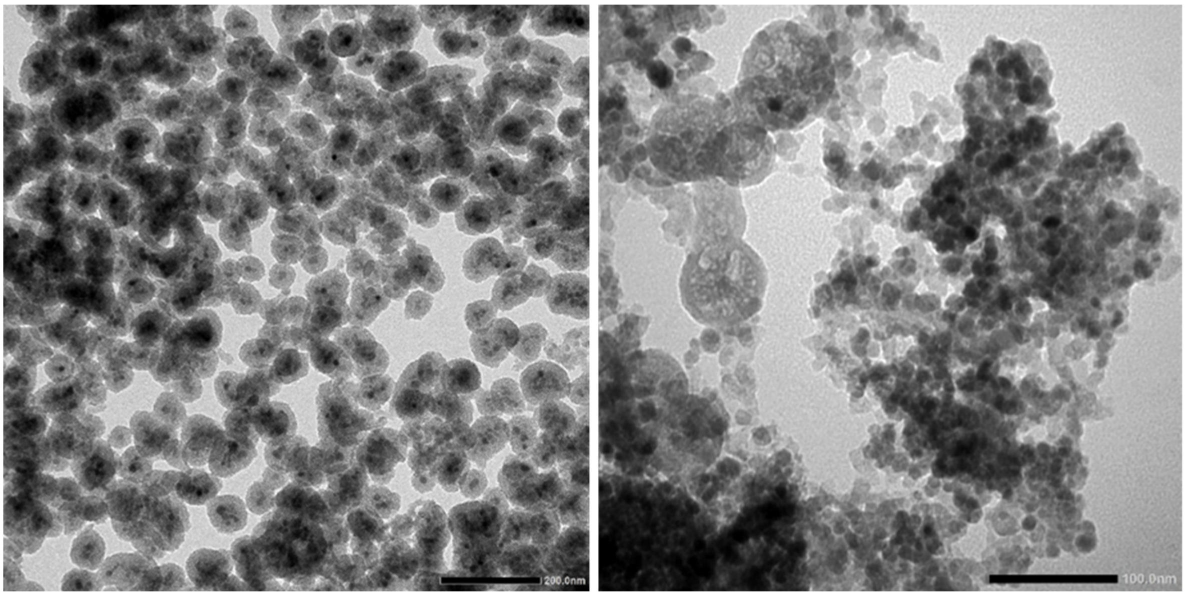

Figure S7. TEM micrographs at 0 (***left***) and 1-month (***right***) NANO3-SH systems from degradation studies in physiological simulated conditions; scale bars at 200 μm and 100 μm, respectively.

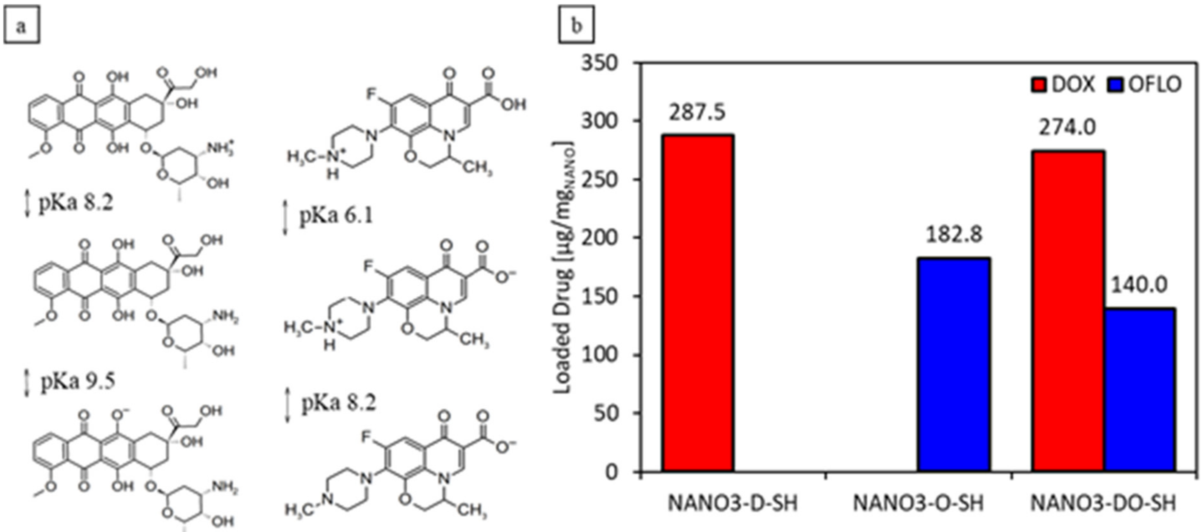

Figure S8. (a) DOX and OFLO pKa in aqueous solutions; **(b)** NANO3-SH systems loading capacities of DOX and OFLO (n=3), in single and dual combinatory formulations

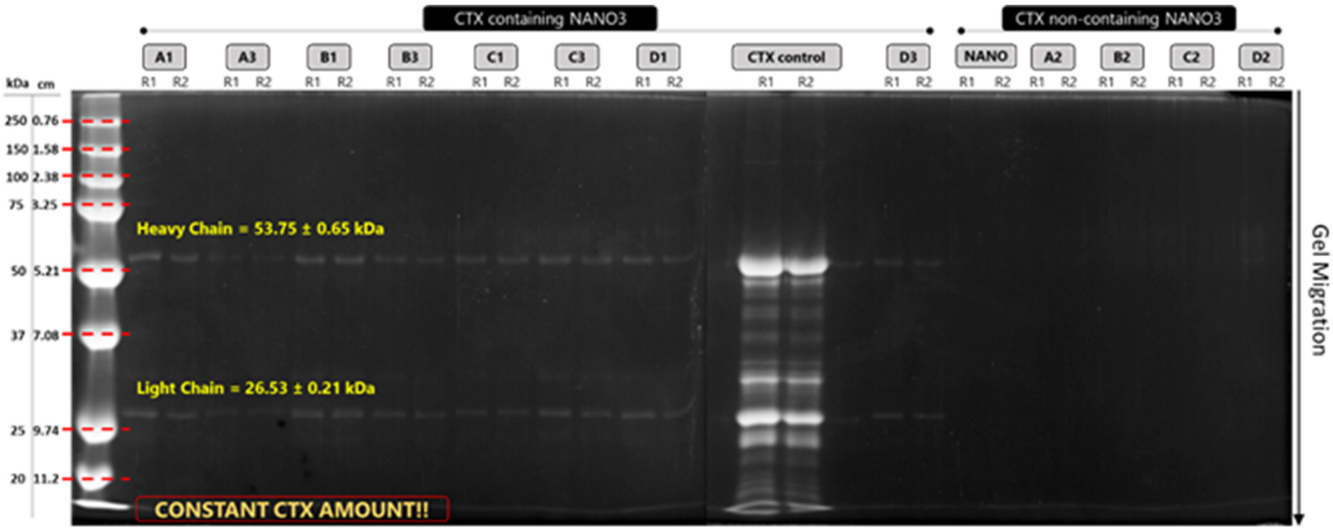

Figure S9. Gel electrophoresis of reduced biofunctionalized NANO3-SH systems loaded with DOX and OFLO (n=2), in single and dual combinatory formulations. Samples: A1= NANO3-CTX, A2 = NANO3-AZM, A3= NANO3-CTX+AZM, B1= NANO3@D-CTX, B2 = NANO3@D-AZM, B3= NANO3@D-CTX+AZM, C1= NANO3@DO-CTX, C2 = NANO3@DO-AZM, C3= NANO3@DO-CTX+AZM, D1= NANO3@O-CTX, D2 = NANO3@O-AZM, D3= NANO3@O-CTX+AZM.
